# RNA terminal uridylyl transferases are druggable vulnerabilities in AML but are dispensable for normal hematopoiesis

**DOI:** 10.1126/sciadv.aec3399

**Published:** 2026-08-12

**Authors:** Christopher Mapperley, Elise Georges, Ali A. Azar, Yuka Kabayama, Hannah Lawson, Iwo Kucinski, Derek George, Joana Campos, Corey Fyfe, Jozef Durko, Wei Y. Chan, Lewis Allen, Babak Jazayeri, Edward Blacker, Louie N. van de Lagemaat, Aurelien Tripp, Theodoros I. Roumeliotis, Giulia Guiducci, Eleanor Herbert, Jasmin Paris, Jyoti Choudhary, George Poulogiannis, Robert M. Campbell, Marcos Morgan, Lovorka Stojic, Folkert J. Van Werven, Douglas Vernimmen, Berthold Göttgens, Dónal O’Carroll, Kamil R. Kranc

**Affiliations:** ^1^ Division of Cancer Biology, The Institute of Cancer Research, London, UK.; ^2^ Barts Cancer Institute, Queen Mary University of London, London, UK.; ^3^ Centre for Regenerative Medicine, University of Edinburgh, Edinburgh, UK.; ^4^ Division of Genome Biology, The Roslin Institute and Royal (Dick) School of Veterinary Studies, University of Edinburgh, Edinburgh, UK.; ^5^ Institute of Cell Biology, University of Edinburgh, Edinburgh, UK.; ^6^ Department of Haematology, Cambridge Stem Cell Institute, Jeffrey Cheah Biomedical Centre, University of Cambridge, Cambridge, UK.; ^7^ Redona Therapeutics, Watertown, MA. USA.; ^8^ The Francis Crick Institute, London, UK.; ^9^ Centre for Genome-Enabled Biology and Medicine, University of Aberdeen, Aberdeen, UK.; ^10^ Division of Cell and Molecular Biology, The Institute of Cancer Research, London, UK.; ^11^ Department of Pathobiology and Population Sciences, Royal Veterinary College, Hatfield, UK.; ^12^ Experimental Histopathology Platform, The Francis Crick Institute, London, UK.; ^13^ The Roslin Institute and Royal (Dick) School of Veterinary Studies, University of Edinburgh, Edinburgh, UK.

## Abstract

Acute myeloid leukemia (AML) is an aggressive hematological malignancy arising from hematopoietic stem and progenitor cells (HSPCs). Current treatments often fail to eradicate AML; therefore, new therapeutic strategies are essential. Here, we reveal that RNA terminal uridylyl transferase enzymes 4 and 7 (TUT4/7) are druggable therapeutic targets, whose genetic deletion suppresses AML growth, induces apoptosis, and improves the survival in leukemic mouse models. Notably, a preclinical TUT4/7 inhibitor promotes cell death in samples from patients with AML and synergizes with venetoclax. Mechanistically, TUT4/7 inactivation suppresses mevalonate pathway gene expression, compromising the cholesterol synthesis pathway. Current AML therapies often cause severe hematopoietic toxicity. Although *Tut4/7* deletion results in inflammatory activation throughout the hematopoietic system, this is permissive to a normal life span and *Tut4/7* deficiency does not compromise HSPC function. Together, these findings identify TUT4/7 as druggable targets, whose inactivation suppresses AML while sparing normal hematopoiesis. In combination with venetoclax, this represents a promising therapeutic strategy.

## INTRODUCTION

Multilineage hematopoiesis relies on a pool of hematopoietic stem and progenitor cells (HSPCs), which maintain lifelong blood cell production while preserving functional capacity and genetic integrity (*1*, *2*). Mutations arising within HSPCs can give rise to a range of myeloid malignancies (*3*), the most aggressive of which is acute myeloid leukemia (AML), a disease associated with a poor prognosis and limited treatment options for the majority of patients (*4*, *5*). Current therapies often fail to eradicate AML and result in substantial toxicity to the hematopoietic system (*6*–*8*), highlighting the need to develop therapies with less toxicity and greater ability to eradicate leukemic cells.

RNA modification pathways have recently emerged as druggable dependencies in AML and other cancers, which can offer opportunities to eradicate malignancies without severe disruption of normal physiology. Although many RNA modifications are essential for AML pathogenesis (*9*–*15*), they are often dispensable for hematopoietic function (*16*, *17*). RNA methylation of adenosine at the N^6^ position (m^6^A), which is critical for the survival and function of AML cells (*9*, *14*, *18*), has generated particular attention as a therapeutic target. Clinical trials targeting RNA modifying enzymes that “write” m^6^A into transcripts are ongoing and producing exciting results across a spectrum of malignancies (*19*–*21*). Here, we report that the writer enzymes of an alternative RNA modification—3′ terminal uridylation—are therapeutically actionable targets in AML and are dispensable for hematopoietic function throughout the life span.

Terminal uridylyl transferases 4 and 7 (TUT4/7) are functionally redundant enzymes encoded by the *ZCCHC11* and *ZCCHC6* genes (hereafter referred to as *TUT4* and *TUT7*) that catalyze the nontemplated addition of uracil residues to the 3′ ends of various RNA species including mRNAs (*22*), microRNAs (miRNAs) (*23*–*27*), and retrotransposons (*28*). While mRNA uridylation marks transcripts for degradation (*22*), miRNA uridylation influences maturation (*24*, *27*), target selection (*26*), and miRNA half-life (*29*). Intriguingly, TUT4/7 are not required for survival (*30*) but are essential to key biological processes including fertility (*30*, *31*), immune function (*32*–*38*), antiviral defense (*39*, *40*), and solid tumor miRNA regulation (*25*, *41*). However, the function and relevance of RNA uridylation in normal and malignant hematopoiesis or cancer pathology in vivo remain unknown.

Here, we demonstrate that RNA uridylation is essential for leukemic propagation and fitness, but not for long-term blood production. Genetic ablation of *Tut4/7* as well as use of a TUT4/7 preclinical inhibitor (*41*) suppresses leukemic growth, induces apoptosis, and improves survival of murine AML models. In contrast, murine hematopoietic-specific *Tut4/7* deletion results in a mild inflammatory phenotype with limited impact at steady state, but with increased age-associated myeloid-biased differentiation and HSPC expansion.

Mechanistically, TUT4/7 loss from AML cells results in a marked reduction in miRNA uridylation, including miRNAs implicated in metabolic regulation, and a transcriptome wide average increase in mRNA half-life that disproportionately affects metabolic transcripts. Genes within the mevalonate pathway, which are essential for AML cell survival via their role in cholesterol synthesis (*42*, *43*) and prenylation (*44*, *45*), are significantly dysregulated by TUT4/7 inactivation, together with related metabolic pathways including fatty acid metabolism.

As these metabolic programs promote AML chemoresistance and represent key therapeutic vulnerabilities (*46*, *47*), their dependence on TUT4/7 activity highlights a tractable therapeutic opportunity. Consistent with this, pharmacological TUT4/7 inhibition synergizes with venetoclax, enhancing antileukemic effect at lower drug concentrations.

Together, our findings highlight a therapeutic window for selective AML targeting with limited impact upon normal hematopoiesis. Given the favorable safety profile of TUT4/7 genetic inactivation, its potent antileukemic activity, its capacity to synergize with existing therapy, and the druggability of RNA modifying enzymes, this study identifies TUT4/7 inhibition as a promising and tractable therapeutic strategy for AML.

## RESULTS

### Leukemic transformation induces TUT4/7 expression and their inactivation compromises AML progression in vivo

Similarly to other modulators of RNA metabolism (*9*, *16*, *48*), *TUT4* and *TUT7* are significantly overexpressed in the leukemic stem cell population when compared to normal hematopoietic stem cells (fig. S1A). To validate this observation experimentally, we cotransduced c-Kit^+^ HSPCs from the bone marrow of mice harboring TUT4 (*Tut4^GFP^*) and TUT7 (*Tut7^GFP^*) green fluorescent protein (GFP) fusion protein reporters (*30*) with retroviruses encoding *Meis1* and *Hoxa9* (hereafter referred to as *Meis1/Hoxa9* cells). These oncogenes are frequently overexpressed in human AML and drive leukemogenesis (*49*, *50*). *Meis1/Hoxa9* overexpression in this context produces leukemic cells able to initiate AML following transplantation (*9*, *51*). GFP expression was significantly elevated following *Meis1/Hoxa9*-driven transformation relative to c-Kit^+^ parental cells (fig. S1B), suggesting that leukemic transformation induces TUT4/7 expression. Furthermore, high *TUT7* expression shows a trend toward association with poor overall survival in patients with AML (*P* value = 0.067) (fig. S1C). Together, these findings, along with clear parallels between TUT4/7 and the m^6^A methyltransferase complex—recently validated as a therapeutic target in AML (*14*, *19*)—prompted us to investigate whether TUT4/7 represents a therapeutic vulnerability in AML.

**Fig. 1.**
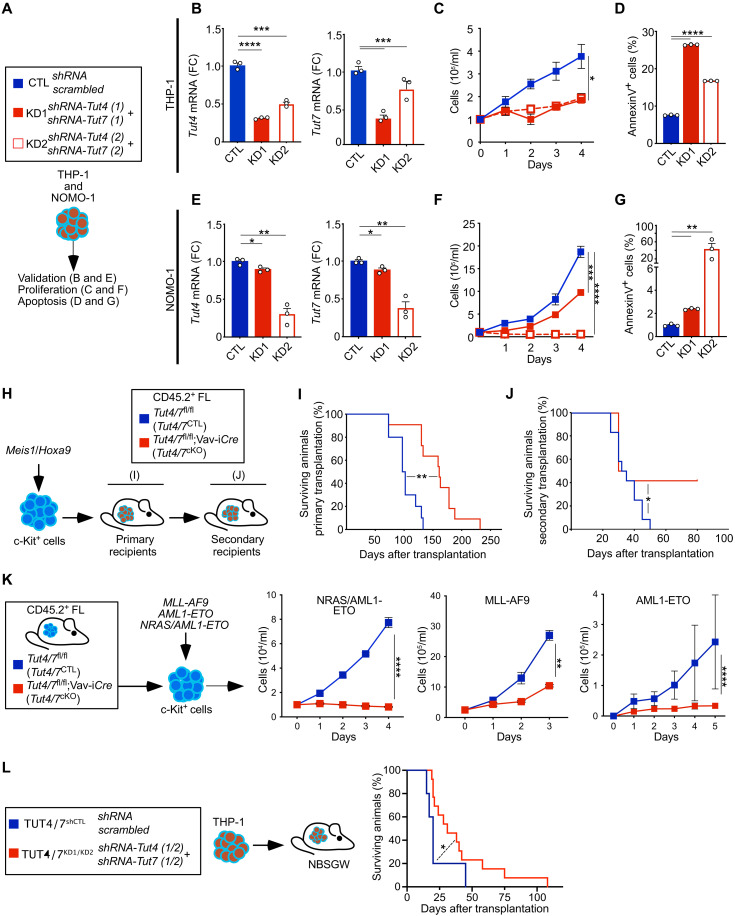
Genetic TUT4/7 inactivation compromises AML. (**A**) *TUT4* and *TUT7* were knocked down in THP-1 and NOMO-1 cells using independent shRNA pairs (KD1 and KD2). Proliferation and apoptosis were compared to cells treated with scrambled shRNA (CTL) (*n* = 3). (**B**) *TUT4* and *TUT7* expression fold change (FC) in THP-1 cells (RT-qPCR), normalized to CTL. Mean ± SEM; ***P < 0.001; ****P < 0.0001 (unpaired *t* test). (**C**) Proliferation of KD1, KD2 and CTL THP-1 cells. Mean ± SEM; *P < 0.05 (unpaired *t* test). (**D**) Percentage of KD1, KD2, and CTL THP-1 cells DAPI^+^Annexin V^+^. Mean ± SEM; ****P < 0.0001 (unpaired *t* test). (**E**) *TUT4* and *TUT7* expression FC in NOMO-1 cells normalized to CTL. Mean ± SEM; *P < 0.05; **P < 0.01 (unpaired *t* test). (**F**) Proliferation of KD1, KD2, and CTL NOMO-1 cells. Mean ± SEM; ***P < 0.001; ****P < 0.0001 (unpaired *t* test). (**G**) Percentage of KD1, KD2, and CTL NOMO-1 cells DAPI^+^Annexin V^+^. Mean ± SEM; **P < 0.01 (unpaired *t* test). (**H**) c-Kit^+^ cells from *Tut4/7^CTL^* and *Tut4/7^cKO^* E14.5 fetal livers were transformed with *Meis1* and *Hoxa9* transgenes (*Meis1/Hoxa9* cells), serially replated, then transplanted into irradiated recipient mice (*n* = 10 to 11). *Meis1/Hoxa9 c*ells were purified from moribund recipient mice and transplanted to irradiated secondary recipients (*n* = 12). (**I**) Survival analysis of primary recipients (**P < 0.01, Kaplan-Meier). (**J**) Survival analysis of secondary recipients (*P < 0.05, Kaplan-Meier). (**K**) Proliferation of c-Kit^+^ cells from *Tut4/7^CTL^* or *Tut4/7^cKO^* mice transformed with *MLL-AF9, AML1-ETO,* or *NRAS/AML1-ETO* oncogenes. Mean ± SEM; **P < 0.01; ****P < 0.0001 (unpaired *t* test) (*n* = 3 to 6). (**L**) Survival analysis of NBSGW mice transplanted with 1600 *Tut4/7^shCTL^* or *Tut4/7^shKD1/KD2^* cells (A). *n* = 13; *P < 0.05 (Kaplan-Meier).

First, we aimed to assess the impact of TUT4/7 inactivation on the fitness of leukemic cells. To begin, we performed dual knockdown (KD) of *TUT4* and *TUT7* in AML cells using two independent short hairpin RNA (shRNA) pairs (KD1 and KD2). Transduction of human THP-1 and NOMO-1 cells with *TUT4/7* shRNA-expressing lentiviruses (Fig. 1, A to G) decreased *TUT4* and *TUT7* expression (Fig. 1, B and E), suppressed AML cell proliferation (Fig. 1, C and F), and induced apoptosis in vitro (Fig. 1, D and G).

To determine the consequences of *Tut4/7* deletion in AML in vivo, we generated conditional *Tut4/7* knockout mice by combining *Tut4^fl^* and *Tut7^fl^* alleles (*30*) with *Vav-iCre* to generate *Tut4^fl/fl^; Tut7^fl/fl^; Vav-iCre^+^* (*Tut4/7^cKO^*) mice, in which *Tut4/7* are deleted within cells of the hematopoietic system (*52*). *Tut4/7^cKO^* and littermate control *Tut4^fl/fl^;Tut7^fl/fl^; Vav-iCre^−^* (*Tut4/7^CTL^*) mice were born at normal Mendelian ratios. Transduction of *Tut4/7^CTL^* and *Tut4/7^cKO^* c-Kit–enriched E14.5 fetal liver cells with retroviruses expressing *Meis1* and *Hoxa9* oncogenes gave rise to murine leukemic cells harboring genetic *Tut4/7* deletion (fig. S1, D and E). Transduced cells were serially replated and transplanted into recipient mice (Fig. 1H). Mice receiving *Tut4/7*-deficient AML cells exhibited a significant delay in disease onset and progression resulting in improved survival compared to recipients of *Tut4/7^CTL^* cells (Fig. 1I), indicating that *Tut4/7* are required for AML initiation and progression.

To assess whether *Tut4/7* deficiency also affected leukemic fitness after disease establishment, AML cells were isolated from the bone marrow of primary recipient mice displaying symptoms of overt leukemia and transplanted to secondary recipients. Mouse survival was also significantly extended in secondary recipients of *Tut4/7^cKO^* cells (Fig. 1J), indicating *Tut4/7* are required for both AML development and maintenance.

To determine whether AML driven by other oncogenes also requires *Tut4/7*, we transformed *Tut4/7^CTL^* and *Tut4/7^cKO^* HSPCs with retroviruses encoding *MLL-AF9, AML1-ETO9a*, and *NRAS/AML1-ETO9a.* Across all oncogenic contexts tested, *Tut4/7*-deficient cells failed to proliferate (Fig. 1K), highlighting a conserved and essential role for *Tut4/7* in leukemic growth irrespective of the initiating oncogenic driver.

Next, we aimed to establish the impact of TUT4/7 inactivation on a human in vivo model of established AML. Thus, we knocked down *TUT4* and *TUT7* in THP-1 cells using shRNAs (Fig. 1B) before transplantation into immunodeficient NBSGW mice. Recipients of *TUT4/7-*knockdown (*TUT4/7^KD^*) THP-1 cells survived significantly longer than recipients of THP-1 cells transduced with a scrambled control shRNA (*TUT4/7^shCTL^*) (Fig. 1L), demonstrating that TUT4/7 activity is required for the progression of established AML.

**Fig. 2.**
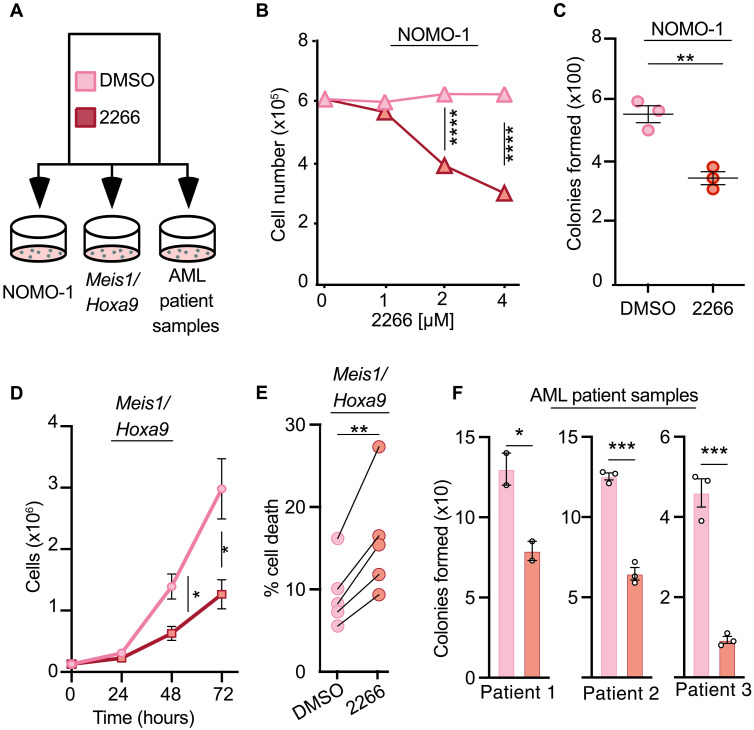
Pharmacological TUT4/7 inactivation compromises AML. (**A**) NOMO-1 cells, murine *Meis1/Hoxa9* cells, and samples from patients with primary AML were treated with 2266 or vehicle control. (**B**) Proliferation of NOMO-1 cells treated with varying concentrations of 2266. Mean ± SEM; ****P < 0.0001 (unpaired *t* test) (*n* = 3). (**C**) NOMO-1 cell colony formation in M3231 Methocult medium with 4 μM 2266 or vehicle control. Data represent mean ± SEM; **P < 0.01 (unpaired *t* test) (*n* = 3). (**D**) *Meis1/Hoxa9* cell proliferation in 4 μM 2266 or vehicle control. Mean ± SEM; *P < 0.05 (unpaired *t* test) (*n* = 4). (**E**) Percentage of *Meis1/Hoxa9* cells Annexin V^+^DAPI^+^, following 48 hours of culture with 4 μM 2266 or vehicle control. Each point represents a biological replicate. **P < 0.01 (paired *t* test) (*n* = 4). (**F**) Colony formation by cells from primary AML patient samples cultured with 10 μM 2266 in H4434 MethoCult medium. Mean ± SEM; *P < 0.05; ***P < 0.001 (unpaired *t* test) (*n* = 2 to 3 replicates per patient sample).

To corroborate genetic approaches and advance toward clinical translation, we evaluated the effects of TS-002266 (2266), a potent TUT4/7 inhibitor able to block miRNA uridylation in vitro with biochemical median inhibitory concentration (IC_50_) values of 69 and 16 nM for TUT4 and TUT7, respectively (*41*). To account for intracellular accessibility and stability, micromolar concentrations were used in cellular experiments. We tested the impact of 2266 on primary murine AML cells, human AML cell lines, and primary samples from AML patients (Fig. 2A). 2266 significantly decreased the proliferation (Fig. 2B) and colony formation (Fig. 2C) of human NOMO-1 cells. Similarly, treatment of murine *Meis1/Hoxa9* transformed cells with 2266 reduced their proliferation (Fig. 2D) and induced apoptosis (Fig. 2E). Moreover, primary AML patient samples treated with 2266 exhibited reduced colony formation capacity (Fig. 2F), indicating that pharmacological TUT4/7 inhibition diminishes the clonogenic potential of leukemic cells.

### Inhibition of TUT4/7 dysregulates expression of crucial metabolic enzymes

**Fig. 3.**
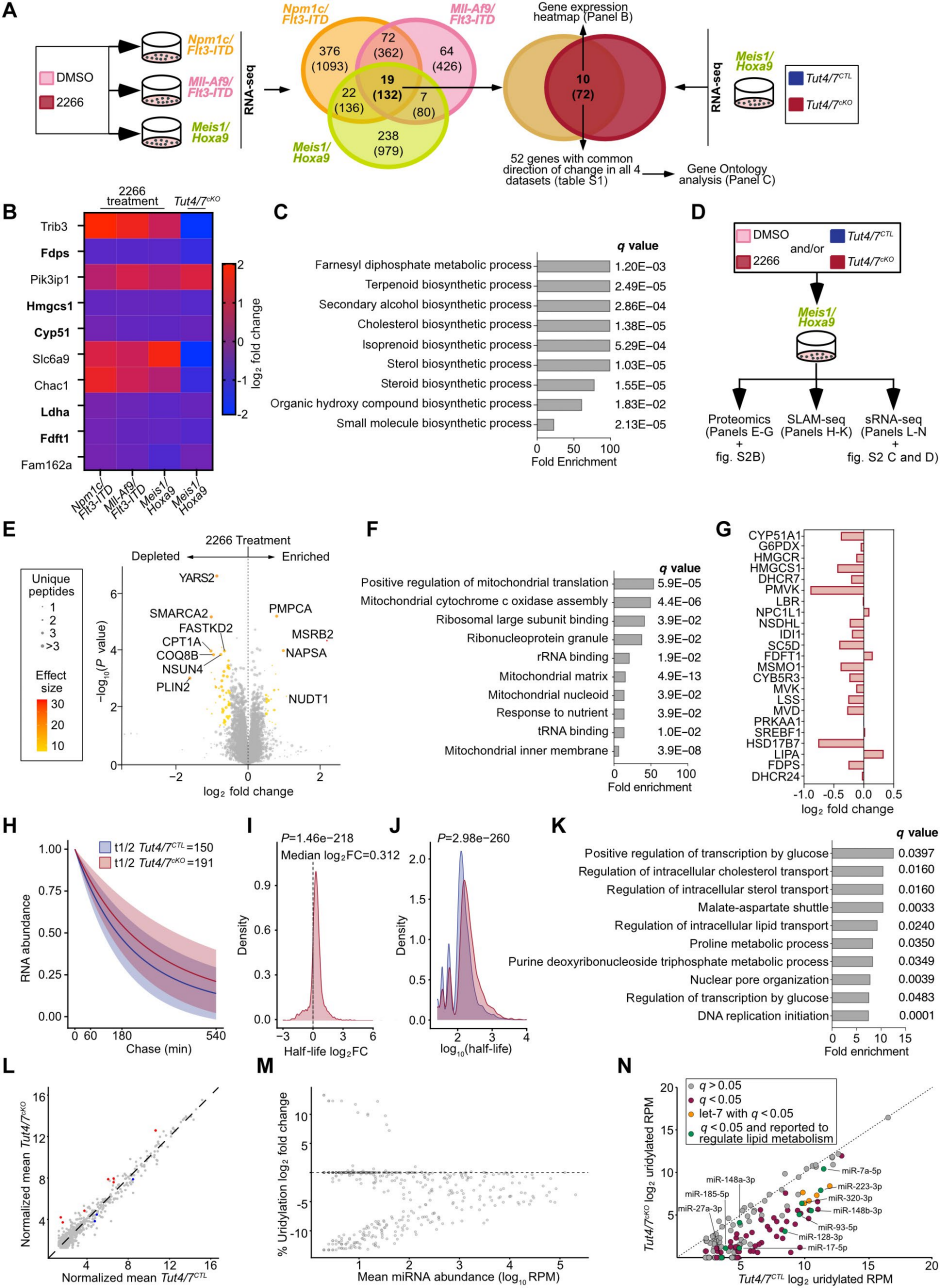
TUT4/7 inactivation dysregulates metabolic gene expression. (**A**) Venn diagram of transcripts dysregulated in *Npm1/ Flt3-ITD* (*n* = 2/3), *Mll-AF9/Flt3-ITD* (*n* = 2/3), and *Meis1/Hoxa9* (*n* = 5) cells treated with 4 μM 2266 versus DMSO (48 hours). Overlapping transcripts were compared to transcripts dysregulated in *Tut4/*7^KO^ (*n* = 3) versus *Tut4/7^CTL^* (*n* = 4) *Meis1/Hoxa9* cells. Number of transcripts significantly dysregulated in overlapping datasets are displayed (numbers above = FDR < 0.05; numbers in brackets = *P* < 0.05). (**B**) Heatmap of log_2_ fold change (FC) in expression of genes significantly dysregulated (FDR < 0.05 independently) in all four datasets. Genes associated with cholesterol synthesis are highlighted in bold. (**C**) Gene Ontology process enrichment, generated from 52 overlapping significantly dysregulated (*P* < 0.05) genes with consistent FC direction in all datasets. (**D**) Proteomics, SLAM-seq, and sRNA-seq analysis of *Meis1/Hoxa9* cells (*n* = 4 to 5) following genetic or pharmacological TUT4/7 inactivation. (**E**) Volcano plot of protein abundance log_2_FC and log_10_ *P* value. Proteins with *P* value <0.05 (pairwise *t* test) are highlighted in yellow. (**F**) Gene Ontology process enrichment, generated from significantly dysregulated proteins (*P* value < 0.05, log_2_FC > 0.5). (**G**) Log_2_FC in abundance of proteins associated with cholesterol biosynthetic process (GO:0006695). (**H**) Mode decay curves for transcript half-life in *Tut4/7^CTL^* and *Tut4/*7^cKO^ *Meis1/Hoxa9* cells. Shaded regions represent the first and third quantile decay curves. (**I**) Histogram of log_2_FC in mRNA transcript half-life. (**J**) Histogram of log_10_-transformed mRNA half-lives. (**K**) Gene Ontology process enrichment, generated from genes with a log_2_FC in half-life of >0.5. (**L**) Normalized mean counts for miRNAs detected in *Tut4/7^CTL^* and *Tut4/7^cKO^ Meis1/Hoxa9* cells; colored points indicate significantly dysregulated miRNAs (FDR < 0.05). (**M**) MA plot of average miRNA abundance (log_10_ RPM) and the log_2_FC in percentage of copies uridylated. (**N**) Normalized read counts of uridylated forms of miRNAs (detected in ≥5/9 samples, with uridylation in ≥3/5 *Tut4/7^CTL^* samples).

Next, we aimed to determine mechanisms by which TUT4/7 inactivation compromises leukemic cells. RNA sequencing (RNA-seq) was performed on 2266-treated murine *Npm1c;Flt3-ITD* leukemic cells, *Mll-Af9;Flt3-ITD* leukemic cells (*53*, *54*), and primary *Meis1/Hoxa9*-transformed leukemic cells, to uncover transcriptional dysregulation resulting from pharmacological inhibition of TUT4/7 across a diverse range of leukemic models. Independently, RNA-seq was conducted on *Meis1/Hoxa9-*transformed *Tut4/7^cKO^* and *Tut4/7^CTL^* cells (Fig. 3A) to identify the effects of genetic *Tut4/7* inactivation. Transcripts significantly dysregulated [false discovery rate (FDR) < 0.05] in all three 2266-treated AML models and in *Tut4/7^cKO^* cells were cross-referenced (Fig. 3A). Overlap in significantly dysregulated genes was significant across all four datasets (table S1). Seventy-two genes exhibited significant dysregulation (*P* < 0.05 in each dataset independently) in all four conditions (fig. S2A). Ten genes were significantly dysregulated by FDR-adjusted *P* value in all four datasets (FDR < 0.05 in each dataset independently) (Fig. 3B). Notably, five of these genes—*Fdps, Hmgcs1, Cyp51, Ldha*, and *Fdft1*—encode key enzymes in the mevalonate pathway, which is responsible for cholesterol and farnesyl synthesis. This consistent down-regulation suggests a mechanistic link between TUT4/7 activity and cholesterol biosynthesis in AML.

To further explore the broader transcriptional effects of TUT4/7 inhibition, we performed Gene Ontology (GO) analysis (*55*) on 52 genes significantly dysregulated in all four datasets displaying consistent directionality in their fold change across each dataset (table S2). Transcripts associated with multiple metabolic pathways were significantly enriched, including farnesyl, cholesterol, and sterol metabolic processes (Fig. 3C), suggesting general dysregulation of the mevalonate pathway.

To determine whether alterations in metabolic gene expression were reflected at the protein level, we performed proteomics on 2266-treated *Meis1/Hoxa9* cells (Fig. 3D). These analyses revealed widespread changes in the abundance of key metabolic proteins (Fig. 3E) including carnitine palmitoyltransferase 1A (CPT1A), the rate-limiting enzyme in mitochondrial fatty acid beta-oxidation. GO analysis of significantly dysregulated proteins revealed widespread changes to metabolic pathways (Fig. 3F and fig. S2B) demonstrating metabolic reprograming following TUT4/7 inactivation.

Confirming results from RNA-seq, the abundance of the majority of proteins associated with the cholesterol biosynthetic process (GO0006695) was decreased at the protein level (Fig. 3G). Thus, TUT4/7 inactivation drives broad-scale alterations in the abundance of key metabolic enzymes.

### TUT4/7 are required for miRNA uridylation and global mRNA stability

Because TUT4/7-mediated RNA uridylation promotes the degradation of modified transcripts (*22*), TUT4/7 inactivation should increase the abundance of their direct targets. Because mevalonate and fatty acid metabolism–related transcripts and proteins are decreased following TUT4/7 inactivation, this is likely an indirect effect. These metabolic alterations indeed could result from TUT4/7-mediated degradation of other mRNAs, or via TUT4/7-mediated uridylation of miRNA. To investigate these possibilities, we performed SLAM-seq [thiol-linked alkylation for the metabolic sequencing of RNA, which measures transcriptome wide changes in mRNA half-life (*56*)] together with small RNA sequencing (sRNA-seq) to assess miRNA abundance and uridylation.

*Tut4/7* deletion significantly extended the average half-life of transcripts from 150 to 191 minutes in *Meis1/Hoxa9* cells (Fig. 3, H and I), representing a 27% average increase in mRNA half-life across the entire transcriptome. Notably, this shift was most predominant among transcripts with the longest half lives in *Tut4/7^CTL^* cells (Fig. 3J), which are typically housekeeping genes such as metabolic enzymes (*57*). GO analysis of transcripts with a log_2_ fold change of >0.5 in *Tut4/7^cKO^ Meis1/Hoxa9* cells revealed significant enrichment for metabolic pathways including positive regulation of transcription by glucose and intracellular transport of cholesterol, sterol, and lipids (Fig. 3K), suggesting that TUT4/7 inactivation stabilizes metabolic enzyme mRNAs. Regulation of cellular metabolism by RNA uridylation aligns with recent reports that m^6^A [another RNA modification associated with mRNA degradation (*58*, *59*)] is a key regulator of glucose and lipid homeostasis (*60*, *61*). Thus, uridylation-mediated RNA degradation may represent a network-level mechanism to regulate the stability and activity of metabolic transcripts.

In addition to regulating mRNA, TUT4/7-mediated uridylation controls the abundance, activity, and target specificity of miRNAs (*23*, *25*, *26*, *34*), many of which are reported to regulate expression of genes within the mevalonate pathway and fatty acid metabolism (*62*–*65*). To determine whether uridylation of these miRNAs was affected in *Tut4/7^cKO^* AML cells, we performed sRNA-seq to assess miRNA abundance and uridylation. Similarly to previous reports (*30*, *31*), *Tut4/7* deletion only resulted in minor changes to miRNA abundance (Fig. 3L); furthermore, there were no notable changes in which miRNAs were most abundant (fig. S2C).

However, *Tut4/7* deletion caused a significant global reduction in miRNA uridylation (Fig. 3M and fig. S3D). Notably, there was substantial loss of uridylation among highly expressed let-7 miRNAs, which can function as key metabolic regulators (*66*–*68*), and miRNAs associated with reported roles in regulation of lipid metabolism and the mevalonate pathway (*69*–*77*) (Fig. 3N). Prominent examples include miR-7 (*71*), miR-148 (*72*), and miR-27a-3p, a known regulator of lipid metabolism and cholesterol homeostasis (*78*–*82*), whose activity and target specificity are modulated by uridylation (*26*). miRNAs repress target transcripts in association with the miRNA-induced silencing complex via translational repression, deadenylation, decapping, and mRNA degradation (*83*). However, their biogenesis, activity, and target repertoire can be modulated by uridylation (*26*, *27*, *84*). Thus, inactivation of TUT4/7 could alter the activity of these key miRNAs enhancing their repression of mevalonate/fatty acid metabolism transcripts.

Furthermore, the alterations to mRNA half-life found in our SLAM-seq data could result not only from loss of direct TUT4/7-mediated degradation but also from alterations in miRNA-mediated degradation. These results suggest a multifaceted mechanism by which TUT4/7 inactivation causes mevalonate pathway dysregulation, potentially involving both miRNA uridylation and direct regulation of metabolic transcripts.

### TUT4/7 inactivation disrupts cholesterol metabolism and sensitizes AML to statin-induced apoptosis

**Fig. 4.**
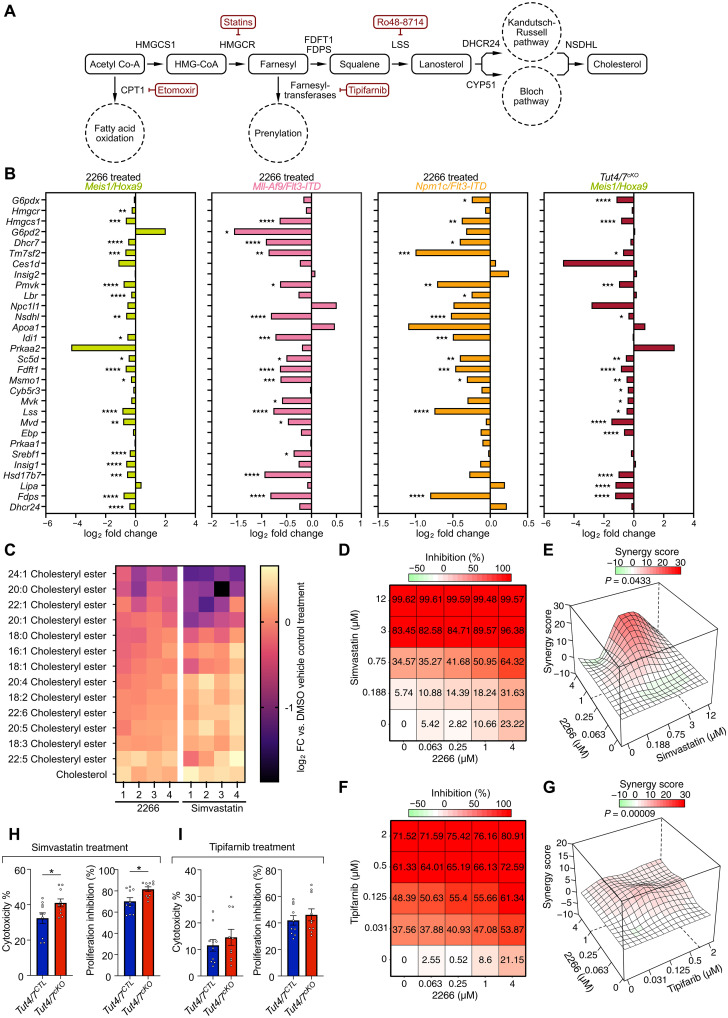
TUT4/7 inactivation dysregulates the mevalonate pathway. (**A**) Schematic of the mevalonate pathway with closely associated fatty acid oxidation and prenylation branches. Acetyl-CoA is metabolized to produce cholesterol and farnesyl. Chemical intermediates are listed in black boxes, enzymes are indicated at each step, and inhibitors targeting enzymes are listed in red. (**B**) Log_2_ fold change (FC) of cholesterol biosynthetic process (GO:0006695)–associated transcripts from RNA-seq described in Fig. 3A (*P < 0.05; **P < 0.01; ***P < 0.001; ****P < 0.0001). (**C**) Heatmap of log_2_FC in cellular cholesterol and cholesteryl esters in *Meis1/Hoxa9* cells treated with 2266 (5 μM) or simvastatin (1 μM), normalized to vehicle control, quantified by targeted metabolomics. (**D**) Percentage growth inhibition of *Meis1/Hoxa9* cells (*n* = 3) by varying concentrations of 2266 and simvastatin after 48 hours, normalized to vehicle-treated cells. (**E**) Synergy score plot of *Meis1/Hoxa9* cells (*n* = 3) treated for 48 hours with varying concentrations of 2266 and simvastatin. (**F**) Percentage growth inhibition of *Meis1/Hoxa9* cells (*n* = 3) by varying concentrations of 2266 and tipifarnib after 48 hours, normalized to vehicle-treated cells. (**G**) Synergy plot of *Meis1/Hoxa9* cells (*n* = 3) treated for 48 hours with varying concentrations of 2266 and tipifarnib. (**H**) Proliferation inhibition and percentage of cells DAPI^+^ in *Tut4/7^CTL^* and *Tut4/7^cKO^ Meis1/Hoxa9* cells treated with 1.5 μM simvastatin for 48 hours, normalized to vehicle treatment. Mean ± SEM, *P < 0.05 (Mann-Whitney *U*). (**I**) Proliferation inhibition and percentage of cells DAPI^+^ in *Tut4/7^CTL^* and *Tut4/7^cKO^ Meis1/Hoxa9* cells treated with 0.5 μM tipifarnib for 48 hours normalized to vehicle treatment. Mean ± SEM (*n* = 10).

Given that multiple independent datasets consistently indicated dysregulation of the mevalonate pathway following TUT4/7 inactivation, we next sought to determine whether this disruption underlies the antileukemic effects of TUT4/7 loss. The mevalonate pathway is responsible for synthesis of the key metabolites farnesyl and cholesterol (*85*) (Fig. 4A). Cholesterol is essential for membrane stability and cellular signaling (*85*, *86*), while the attachment of farnesyl to proteins (i.e., prenylation (*87*)) is a key posttranslational modification that is essential for protein localization to cellular membranes and is particularly associated with RAS pathway activity (*45*). Statins inhibit 3-hydroxy-3-methylglutaryl-CoA reductase (HMGCR), an essential enzyme in the mevalonate pathway upstream of both farnesyl and cholesterol production (Fig. 4A), and their use is associated with improved outcomes in various malignancies (*42*, *88*, *89*). Extensive investigation of mevalonate pathway inhibitors has demonstrated their potential in a variety of cancers (*43*, *45*) including AML (*42*, *90*), resulting in clinical trials across a spectrum of blood malignancies (*91*–*95*).

Therefore, we aimed to determine whether the impact of *Tut4/7* deletion on AML was caused by mevalonate pathway dysregulation. First, we examined the expression of all genes associated with the cholesterol biosynthesis pathway (GO:0045540) following 2266 treatment or genetic deletion of *Tut4/7*. Across all four datasets, targeting TUT4/7 significantly down-regulated the majority of cholesterol biosynthesis–associated transcripts (Fig. 4B), suggesting conserved mevalonate dysregulation across all AML models tested.

Because *Tut4/7* deletion dysregulates the mevalonate pathway, it was hypothesized that pharmacologic TUT4/7 inactivation via 2266 would produce metabolic effects similar to those of statin treatment. To test this, we performed targeted metabolomics in *Meis1/Hoxa9* cells treated with 2266 or the potent HMGCR inhibitor simvastatin (*44*). Treatment with 2266 closely recapitulated the metabolic impact of simvastatin, producing a consistent and widespread reduction in cholesteryl ester abundance (Fig. 4C and fig. S2E). This pattern is consistent with increased mobilization of cholesteryl ester stores, likely reflecting a compensatory response by leukemic cells to maintain intracellular cholesterol levels following inhibition of biosynthesis.

We next aimed to determine whether the antileukemic effect of TUT4/7 inhibition resulted from compromised mevalonate pathway activity and probe whether this is dependent on the cholesterol synthesis or prenylation pathway branches. Therefore, we combined 2266 treatment with simvastatin (targeting the mevalonate pathway upstream of farnesyl production), tipifarnib (targeting prenylation post–farnesyl production), RO48-8071 (targeting cholesterol synthesis downstream of farnesyl production) (*96*), and etomoxir (targeting fatty acid metabolism) (*97*) (Fig. 4A). This allowed us to probe the mevalonate pathway upstream and downstream of prenylation, prenylation itself, and the closely related fatty acid oxidation pathway, to determine whether loss of TUT4/7 dysregulated overall lipid metabolism. Simvastatin (Fig. 4, D and E) and tipifarnib (Fig. 4, F and G) synergized with 2266, significantly affecting the growth of *Meis1/Hoxa9*-transformed cells. In contrast, RO48-8071 (fig. S2F) and etomoxir (fig. S2G) had no synergy with TUT4/7 inhibition. Simvastatin (Fig. 4H) but not tipifarnib (Fig. 4I) had a significantly greater cytotoxic and growth inhibitory effect upon *Tut4/7^cKO^* than *Tut4/7^CTL^* cells. Collectively, this suggests that TUT4/7 inhibition synergizes with compounds targeting the mevalonate pathway, specifically those inhibiting prenylation, but that blockade of cholesterol production downstream of prenylation or overall lipid metabolism does not synergize with TUT4/7 inhibition. Given previous clinical trial data for statins and prenylation inhibitors for treatment of blood malignancies (*91*, *92*, *94*), combinatorial targeting with TUT4/7 inhibition offers a promising alternative therapeutic approach.

### TUT4/7 inhibition sensitizes AML cells to venetoclax

Statins enhance the efficacy of the BCL2 inhibitor venetoclax in a variety of blood cancers, which rely on prenylation to avoid therapy-induced apoptosis (*98*), resulting in ongoing clinical trials combining venetoclax with pitavastatin (*91*). Furthermore, we noted that loss of TUT4/7 activity increased *Bcl2* expression (fig. S2H) across all four of our RNA-seq datasets (significantly in *Tut4/7^cKO^ Meis1/Hoxa9* cells and 2266 treated *Npm1c;Flt3-ITD* cells), suggesting that AML cells may use BCL2 to evade apoptosis driven by TUT4/7 inactivation. Given this observation, and because TUT4/7 inhibition down-regulates mevalonate pathway gene expression and synergizes with prenylation blockade, we hypothesized that TUT4/7 inhibition might also synergize with venetoclax.

**Fig. 5.**
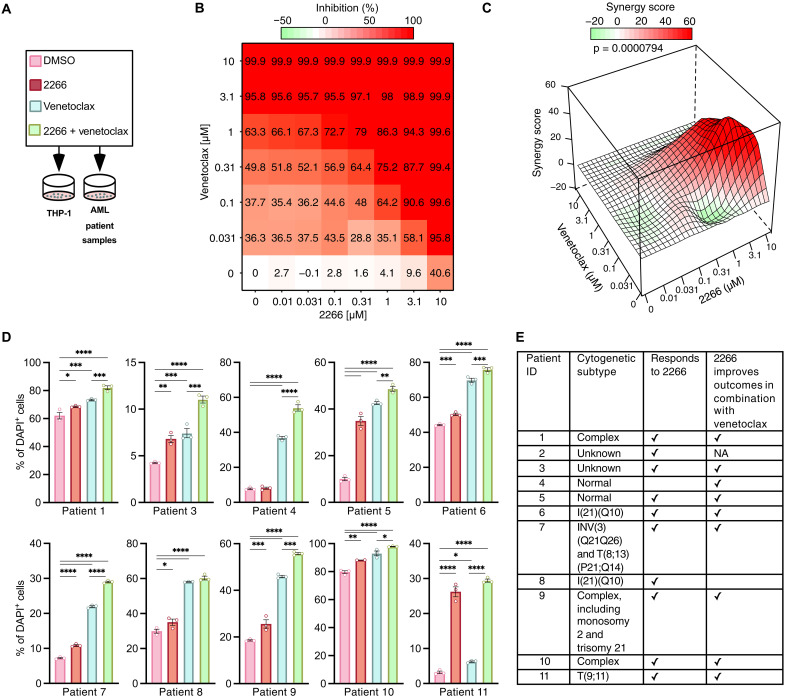
TUT4/7 inhibition synergizes with venetoclax. (**A**) Experimental design; THP-1 cells and cells from primary AML patient samples were treated with DMSO vehicle control, 2266, venetoclax, or a combination of 2266 and venetoclax. (**B**) Growth inhibition matrix for THP-1 cells grown in 2266 and venetoclax for 48 hours, normalized to vehicle-treated cells. (**C**) Bliss synergy score plot of THP-1 cells grown for 48 hours in varying concentrations of venetoclax and 2266. (**D**) Cell death (% of cells DAPI^+^) in samples from patients with AML after 48 hours of culture with DMSO vehicle control, 10 μM 2266, 1 μM venetoclax, or a combination of 10 μM 2266 and 1 μM venetoclax. Data represent mean ± SEM; *P < 0.05; **P < 0.01; ***P < 0.001; ****P < 0.0001 (one-way ANOVA) (*n* = 3). (**E**) Table describing cytogenetic subtypes of samples from patients with AML and their response to 2266 alone or in combination with venetoclax. Tick indicates significant response to 2266 in comparison to DMSO control or venetoclax alone (*P* < 0.05, one-way ANOVA) (*n* = 3).

THP-1 cells and samples from patients with primary AML were treated with 2266 and venetoclax, either alone or in combination (Fig. 5A). In THP-1 cells, 2266 significantly enhanced the cytotoxic effects of venetoclax (Fig. 5, B and C) synergizing to induce cell death at a lower concentration. Notably, cotreatment with 2266 reduced the IC_50_ of venetoclax from 0.31 to 0.1 μM in vitro (Fig. 5B), indicating increased sensitivity.

To assess the potential of this combination in a clinically relevant setting, a panel of 10 primary AML patient samples were treated with 2266 alone or in combination with venetoclax. 2266 alone induced a significant increase in apoptosis in 9 of 10 samples. Notably, the combination of 2266 with venetoclax provided additional benefit in 9 of 10 samples, although in this context, the effect was additive rather than multiplicative, which may reflect the intrinsically higher level of baseline apoptosis within samples from patients with AML (Fig. 5D). 2266’s cytotoxic effect was independent of specific cytogenetic subtypes (Fig. 5E). These findings support the therapeutic potential of TUT4/7 inhibition, either as a monotherapy or in combination with venetoclax.

### Genetic or pharmacological TUT4/7 inactivation does not derail normal hematopoietic function

Efficacious AML therapies must achieve selective toxicity against malignant cells while sparing normal hematopoietic function. Current AML treatments often cause debilitating side effects, and many promising therapies are ultimately rendered nonviable due to their hematopoietic toxicity (*6*, *8*). Therefore, we investigated the impact of targeting TUT4/7 on normal hematopoiesis.

**Fig. 6.**
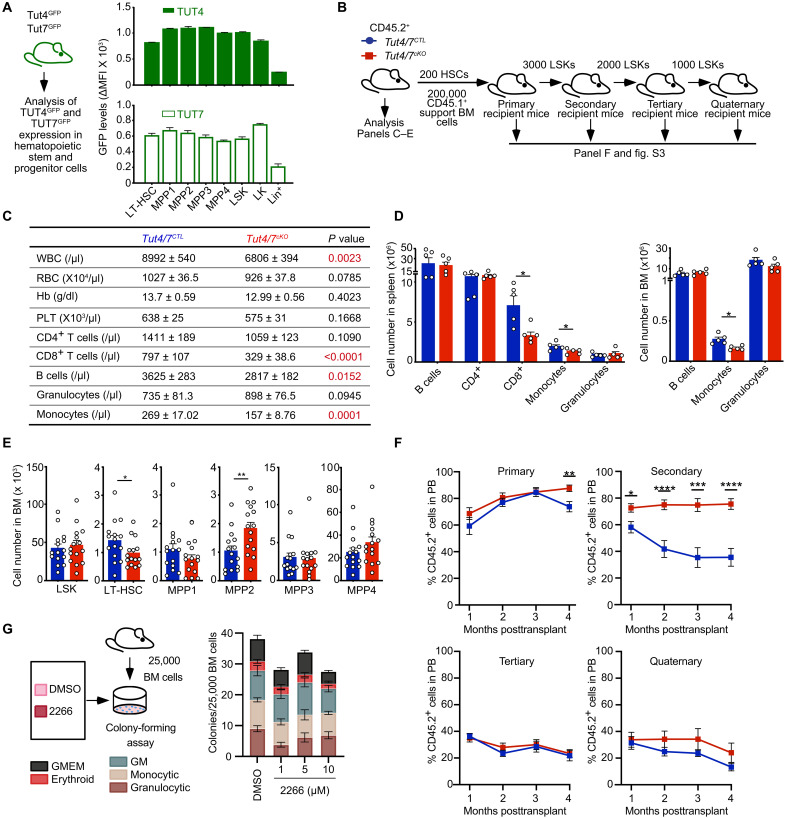
Genetic ablation of *Tut4/7* does not derail normal hematopoiesis. (**A**) TUT4-GFP and TUT7-GFP expression in long-term hematopoietic stem cells (LT-HSCs); multipotent progenitor cell (MPP) populations 1, 2, 3, and 4; LSK^+^ cells; LK^+^ cells; and lineage^+^ (Lin^+^) cells from *Tut4^GFP/GFP^* and *Tut7^GFP/GFP^* mice (*n* = 3). Results normalized to C57BL/6 controls. Immunophenotypes are described in table S5. (**B**) The composition of the hematopoietic system in *Tut4/7^CTL^* and *Tut4/7^cKO^* mice was analyzed. Furthermore, CD45.2^+^ HSCs purified from *Tut4/7^CTL^* and *Tut4/7^cKO^* mice were transplanted into irradiated recipient CD45.1/2^+^ mice with 200,000 CD45.1^+^ bone marrow cells. This procedure was serially repeated with LSK^+^ cells purified from previous recipient mice to produce secondary, tertiary, and quaternary transplantations. (**C**) Counts of white blood cells (WBC), red blood cells (RBC), hemoglobin (Hb), platelets (PLT), CD4^+^ T cells, CD8^+^ T cells, CD19^+^B220^+^ B cells, CD11b^+^GR1^+^ granulocytes, and CD11b^+^GR1^−^ monocytes in the peripheral blood of *Tut4/7^CTL^* and *Tut4/7^cKO^* mice. Significantly dysregulated parameters (*P* < 0.05 Mann-Whitney *U*) are highlighted in red (*n* = 5). (**D**) *Tut4/7^CTL^* and *Tut4/7^cKO^* splenic and bone marrow (tibia + femur) differentiated cell quantification. Mean ± SEM; *P < 0.05 (Mann-Whitney *U*) (*n* = 5). (**E**) *Tut4/7^CTL^* and *Tut4/7^cKO^* bone marrow hematopoietic stem and progenitor cell quantification. Mean ± SEM; *P < 0.05; **P < 0.01 (Mann-Whitney *U*) (*n* = 15). (**F**) Percentage of CD45.2^+^ *Tut4/7^CTL^* and *Tut4/7^cKO^* cells in the peripheral blood of primary, secondary, tertiary, and quaternary transplanted mice. Mean ± SEM; *P < 0.05; **P < 0.01; ***P < 0.001; ****P < 0.0001 (Mann-Whitney *U*, *n* = 10 to 20). (**G**) Murine bone marrow cells were plated into M3434 Methocult medium containing 2266 or vehicle control. Formation of monocytic, granulocytic, GM (granulocytic and monocytic), erythroid, or GMEM (granulocytic, monocytic and erythroid) hematopoietic colonies after 10 days. Mean ± SEM for each colony type (*n* = 4).

First, we used TUT4 and TUT7 GFP reporter mice (*Tut4^GFP^* and *Tut7^GFP^*) (*30*) to determine TUT4/7 expression at different levels of the hematopoietic hierarchy. *Tut4^GFP^* and *Tut7^GFP^* were highly expressed in long-term hematopoietic stem cells (LT-HSCs), which retain the ability to self-renew through the life span and give rise to all blood lineages (*99*, *100*). TUT4/7 expression was highest in multipotent progenitor cells (MPPs) within the lineage^−^Sca-1^+^c-Kit^+^ (LSK) compartment. MPPs can produce all blood lineages but lack the long-term self-renewal potential of HSCs (*99*, *100*). Cycling, lineage committed hematopoietic progenitor cells—lineage^−^Sca-1^−^c-Kit^+^ (LK)—displayed high TUT4/7 expression, whereas expression was low in mature lineage^+^ differentiated blood cells (Fig. 6A).

Next, we examined the hematopoietic system of mice harboring hematopoietic-specific *Tut4/7* deletion (*Tut4/7^cKO^*) (Fig. 6B). Peripheral blood analysis of *Tut4/7^cKO^* mice revealed a significant deficit in CD8^+^ T cells, B cells, and monocytes, resulting in leukopenia (Fig. 6C). In the spleen and the bone marrow, the immune composition of *Tut4/7^cKO^* mice was largely normal, apart from small decreases in the number of monocytes and CD8^+^ T cells (Fig. 6D). LT-HSCs were depleted in the bone marrow of *Tut4/7^cKO^* mice, while the myeloid/megakaryocyte biased multipotent progenitor cell 2 (MPP2) population was expanded (Fig. 6E).

To evaluate whether loss of *Tut4/7* affected hematopoietic function, we assessed the multilineage reconstitution capacity of *Tut4/7^cKO^* versus *Tut4/7^CTL^* HSCs transplanted into irradiated primary recipient mice (Fig. 6B). *Tut4/7^cKO^* HSCs showed an increased ability to engraft following transplantation (Fig. 6F) and did not affect the ability of HSCs to reconstitute the stem cell compartment or form all blood lineages (fig. S3, A to C). Furthermore, *Tut4/7^cKO^* HSCs were able to continually reconstitute hematopoiesis when serially transplanted into secondary, tertiary, and quaternary recipients (Fig. 6F and fig. S3, D to I). Notably, in secondary transplantation, *Tut4/7^cKO^* HSCs display significantly greater engraftment than *Tut4/7^CTL^* counterparts (Fig. 6F and fig. S3, D and E). Therefore, loss of *Tut4/7* may be beneficial for HSC function in the medium term, without detrimental effects on long-term HSC self-renewal over a 16-month period.

Because genetic *Tut4/7* ablation does not affect hematopoietic fitness at steady state or upon transplantation, we sought to validate these results using pharmacological TUT4/7 inactivation. Across a range of concentrations, 2266 did not significantly reduce colony formation by mouse bone marrow cells (Fig. 6G). In addition, 2266 did not impair the ability of HSPCs to differentiate in vitro (Fig. 6G). Therefore, TUT4/7 pharmacological inhibition selectively compromises AML cells without affecting HSPC function in vitro.

### *Tut4/7* deficiency dysregulates the multipotent progenitor cell pool with age resulting in extramedullary hematopoiesis and a myeloid bias

**Fig. 7.**
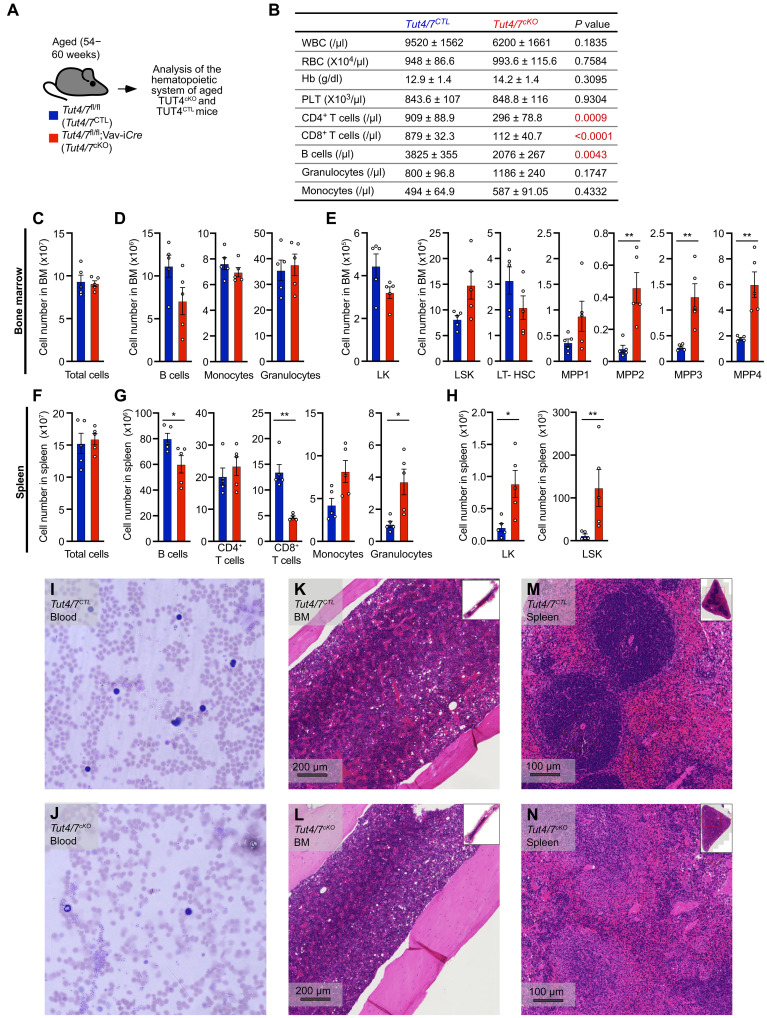
Old *Tut4/7-*deficient mice display expansion of hematopoietic progenitor cells and myeloid skewing. (**A**) Experimental design; the hematopoietic system of aged *Tut4/7^CTL^* and *Tut4/7^cKO^* mice was analyzed at 54 to 60 weeks of age (*n* = 5). (**B**) Counts of white blood cells (WBC), red blood cells (RBC), hemoglobin (Hb), platelets (PLT), CD4^+^ T cells, CD8^+^ T cells, CD19^+^B220^+^ B cells, CD11b^+^GR1^+^ granulocytes, and CD11b^+^GR1^−^ monocytes in the peripheral blood of aged *Tut4/7^CTL^* and *Tut4/7^cKO^* mice. Significantly dysregulated parameters are highlighted in red (*P* < 0.05 Mann-Whitney *U* test). Mean ± SEM. (**C** to **E**) Quantification of the total cellularity (C), differentiated cells (D), and hematopoietic stem and progenitor cells (E) in the bone marrow (tibia + femur) of aged *Tut4/7^CTL^* and *Tut4/7^cKO^* mice. Mean ± SEM (Mann-Whitney *U*). (**F** to **H**) Quantification of the total cellularity (F), differentiated cells (G), and hematopoietic stem and progenitor cells (H) in the spleens of aged *Tut4/7^CTL^* and *Tut4/7^cKO^* mice. Mean ± SEM; *P < 0.05; **P < 0.01 (Mann-Whitney *U*). (**I** and **J**) Representative images of Wright-Giemsa–stained blood smears from aged *Tut4/7^CTL^* (I) and *Tut4/7^cKO^* (J) mice. (**K** and **L**) Representative sections of H&E-stained bone marrow from aged *Tut4/7^CTL^* (K) and *Tut4/7^cKO^* (L) mice. (**M** and **N**) Representative sections of H&E-stained spleen from aged *Tut4/7^CTL^* (M) and *Tut4/7^cKO^* (N) mice.

Inactivation of other RNA modification pathways including m^6^A and pseudouridylation has previously been found to influence hematopoietic aging and can cause premature stem cell exhaustion (*16*, *101*, *102*). To investigate whether the same is true of *Tut4/7^cKO^*, we aged mice and analyzed the composition of the hematopoietic system (Fig. 7A). Both *Tut4/7^CTL^* and *Tut4/7^cKO^* mice reached 54 to 60 weeks of age without overt pathology or any difference in overall survival. Therefore, similarly to tamoxifen-inducible *R26Cre^ERT^*-mediated *Tut4/7* deletion (*30*), hematopoiesis-specific *Tut4/7* deletion is permissive and does not affect overall life span. However, CD4^+^ T cells, CD8^+^ T cells, and B cells were markedly depleted in the peripheral blood of aged *Tut4/7^cKO^* mice (Fig. 7B), indicating an inability to adequately produce or maintain the lymphoid lineage.

Aged *Tut4/7^cKO^* mice displayed no significant changes to the total cellularity of the bone marrow (Fig. 7C), or to total numbers of differentiated cell populations within the bone marrow (Fig. 7D). However, myelo-erythroid biased multipotent progenitor 2 cells (MPP2), myeloid-biased multipotent progenitor 3 cells (MPP3), and lymphoid biased multipotent progenitor 4 cells (MPP4) cells (*99*, *100*) were all significantly expanded (Fig. 7E), indicating broad expansion of the hematopoietic progenitor cell pool. Splenic total cellularity was unaffected by *Tut4/7^cKO^* (Fig. 7F); however, splenic immune composition mirrored the blood and bone marrow with a deficit in lymphoid cell numbers (B cells, CD4^+^ T cells, and CD8^+^ T cells) and myeloid expansion (monocytes and granulocytes) (Fig. 7G). Intriguingly, HSPCs within both the LK and LSK populations were greatly enriched in the spleens of *Tut4/7^cKO^* mice (Fig. 7H), indicating extramedullary hematopoiesis.

In patients, splenic HSPC expansion is indicative of hematopathology or malignancy and can result from insufficient stem and progenitor cell function in the bone marrow, an inflammatory or fibrotic bone marrow microenvironment, or the emergence of malignant cells with excessive self-renewal potential (*103*). Therefore, we aimed to determine whether loss of *Tut4/7* induces a premalignant phenotype by analyzing blood smears (Fig. 7, I and J) and histological samples from the bone marrow (Fig. 7, K and L) and spleen (Fig. 7, M and N) of aged *Tut4/7^CTL^* and *Tut4/7^cKO^* mice. While spleens from *Tut4/7^cKO^* mice were distorted, with reduced white pulp area and cellularity, we found no evidence of dysplasia, bone marrow fibrosis, malignant transformation, or gross changes to the bone marrow structure.

We conclude that in aged mice, *Tut4/7* deletion dysregulates the hematopoietic progenitor cell pool, resulting in excessive expansion, splenic infiltration, and myeloid proliferation. Crucially, adverse effects of TUT4/7 inactivation are relatively mild and only emerge in year-old mice. These findings highlight a therapeutic window for targeting TUT4/7 without adverse effects on hematopoiesis.

### Pro-inflammatory signaling is activated in *Tut4/7*-deficient HSPCs

**Fig. 8.**
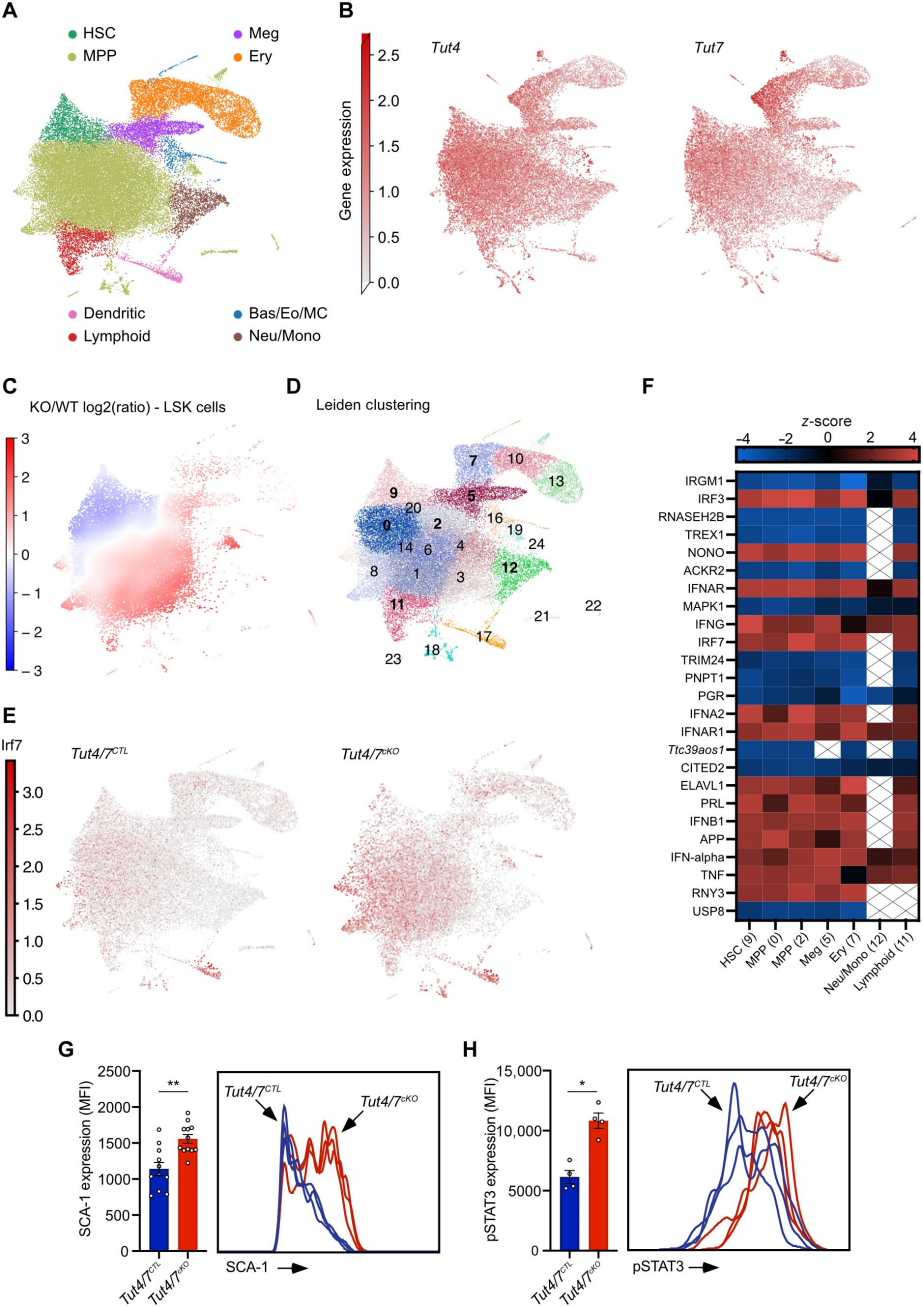
*Tut4/7* ablation induces inflammatory activation in hematopoietic stem and progenitor cells. (**A**) UMAP projection of the single-cell RNA-seq (scRNA-seq) landscape generated from sorted hematopoietic stem and progenitor cells (LSKs and LKs) indicating the location of cells classified as hematopoietic stem cells (HSC), multipotent progenitor cells (MPP), or progenitor cells with differentiation potential biased toward the megakaryocyte (Meg), erythroid (Ery), dendritic, lymphoid, basophilic/eosinophil/mast cell, or neutrophil/monocyte lineages. (**B**) Expression of *Tut4* and *Tut7* determined from scRNA-seq. (**C**) Relative enrichment (log_2_ ratio) of *Tut4/7^cKO^* LSK cells compared to *Tut4/7^CTL^* LSK cells representing enrichment or depletion of differential cell identities from scRNA-seq. (**D**) Leiden clustering of scRNA-seq from combined *Tut4/7^CTL^* and *Tut4/7^cKO^* samples. (**E**) Expression of *Irf7* in hematopoietic cells from *Tut4/7^CTL^* and *Tut4/7^cKO^* mice. (**F**) Heatmap of *z*-scores derived from ingenuity pathway analysis (IPA) upstream regulator analysis of Leiden clusters representing HSCs (9), MPPs (0), MPPs (2), and HSPCs biased toward megakaryocytic (5), erythroid (7), neutrophil/monocyte (12), and lymphoid (11) differentiation. Pathways where expression of target genes was not detected in specified clusters are left blank. (**G**) SCA-1 expression in *Tut4/7^CTL^* (*n* = 11) and *Tut4/7^cKO^* (*n* = 12) HSCs (LSK^+^, CD48^−^, CD150^+^). (**H**) Expression of phosphorylated STAT3 in *Tut4/7^CTL^* and *Tut4/7^cKO^* HSCs (LSK^+^, CD48^−^, CD150^+^) (*n* = 4).

To reveal the transcriptional consequences of *Tut4/7* deletion in hematopoiesis and to understand how this causes HSPC expansion and myeloid biased differentiation with age, we performed 10× single-cell RNA sequencing (scRNA-seq) on sorted *Tut4/7^CTL^* and *Tut4/7^cKO^* hematopoietic stem and progenitor LK and LSK populations from 8- to 12-week-old mice (fig. S4A). Uniform Manifold Approximation and Projection (UMAP) was used for dimensionality reduction and cell lineages were manually annotated across all datasets (Fig. 8A). *Tut4* and *Tut7* expression across the hematopoietic hierarchy (Fig. 8B) was consistent with flow cytometry results (Fig. 6A), with the highest expression levels in MPPs, lower levels in HSCs, and progressively decreasing expression along differentiation trajectories.

Cell abundance analysis of LSK (Fig. 8C) and LK (fig. S4B) cell compartments also corroborated flow cytometric results (Fig. 6E). Within the LSK compartment of *Tut4/7^cKO^* mice, primitive LT-HSCs were depleted and more immature progenitor cells were enriched (Fig. 8C). No additional or aberrant populations distinct from those in *Tut4/7^CTL^* samples were identified in *Tut4/7^cKO^* mice.

Leiden clustering and expression of canonical marker genes identified cell clusters representative of all known hematopoietic populations (Fig. 8D). Gene expression of cells within these clusters was pseudo-bulked to perform differential expression analysis highlighting variation in genetic dysregulation by cluster (fig. S4C). Notably, gene set enrichment analysis (GSEA) revealed significant enrichment of inflammatory response genes associated with the interferon alpha and gamma responses in all but one cluster (table S3), and the key inflammatory mediator interferon regulatory factor 7 (*Irf7*), which acts downstream of nucleic acid sensing pathways (*104*), was highly enriched across the hematopoietic hierarchy (Fig. 8E). Thus, key pro-inflammatory genes are up-regulated throughout the hematopoietic hierarchy in *Tut4/7^cKO^* mice.

To further explore regulation of inflammation within *Tut4/7-*deficient hematopoiesis, we focused on Leiden clusters reflective of different populations across the hematopoietic hierarchy: cluster 9 (HSCs), cluster 0 (MPPs), cluster 2 (MPPs), cluster 5 (megakaryocyte progenitors), cluster 7 (erythroid progenitors), cluster 12 (neutrophilic/monocytic progenitors), and cluster 11 (lymphoid progenitors). Ingenuity pathway analysis identified up-regulation of transcripts downstream of multiple key inflammatory mediators across the hierarchy, including targets of IRF7, IRF3, INFAR, and IFNG2, while targets of anti-inflammatory mediator IRGM1 were down-regulated. In addition, dysregulation of genes downstream of many RNA processing pathways including RNASEH2B, TREX1, and ELAVL1 was observed (Fig. 8F and fig. S4D). RNASEH2B (*105*) and TREX1 (*106*) are nucleases required for the degradation of harmful nucleic acid species, which otherwise form anomalous structures resulting in a cell-intrinsic innate immune response (*107*). *RNASEH2B* and *TREX1* mutations result in the systemic autoimmune disease *Aicardi*-Goutières syndrome (*105*, *106*), which is also associated with mutations in the RNA modifying enzyme *ADAR1.* Thus, hematopoietic *Tut4/7* deletion phenocopies the systemic inflammatory activation associated with loss of other RNA processing factors (*16*, *108*, *109*).

Chronic inflammatory activation within the hematopoietic system is known to drive HSPC expansion, myeloid bias, and extramedullary hematopoiesis, particularly with age (*110*). Given that loss of *Tut4/7* induces such phenotypes, we sought to confirm that inflammatory pathways are activated within the HSPC compartment. We found that *Tut4/7^cKO^* HSCs have elevated expression of cell surface SCA-1 (Fig. 8G), commonly used as a proxy of type I interferon signaling (*111*). Furthermore, phosphorylation of the key inflammatory mediator STAT3 was significantly increased in *Tut4/7^cKO^* HSCs, implying intrinsic activation of its inflammatory signal transduction program (Fig. 8H).

Thus, loss of *Tut4/7* induces cell-intrinsic inflammatory activation across the hematopoietic system, leading to myeloid-biased differentiation, HSPC expansion, and extramedullary hematopoiesis. However, these phenotypes are minimal or absent in young mice, and independent reports indicate that inducible *Tut4/7* deletion is well tolerated (*30*). Thus, TUT4/7 inactivation exerts differential impacts on normal versus malignant hematopoiesis.

To understand mechanisms underlying this selectivity, we compared the transcriptional impact of TUT4/7 deletion on normal versus malignant hematopoiesis. GSEA revealed that TUT4/7 inactivation exerts opposing transcriptional impacts (fig. S4E). For example, MYC Targets and Oxidative Phosphorylation hallmarks were down-regulated in leukemic cells but up-regulated in normal hematopoietic cells. Inflammatory pathways such as the Interferon Alpha and Gamma Response are elevated in multiple normal haemopoietic cell types but were not significantly affected by TUT4/7 inactivation in leukemic cells. Fatty Acid Metabolism was significantly down-regulated in leukemic cells but was unaffected by *Tut4/7* deletion in normal hematopoietic subsets (fig. S4E). Thus, TUT4/7 inactivation elicits fundamentally divergent transcriptional responses in normal and leukemic hematopoiesis, with selective repression of metabolic programs in malignant cells. Together, these findings provide evidence that TUT4/7 inhibition selectively targets AML with minimal impact on normal hematopoiesis.

## DISCUSSION

**Fig. 9.**
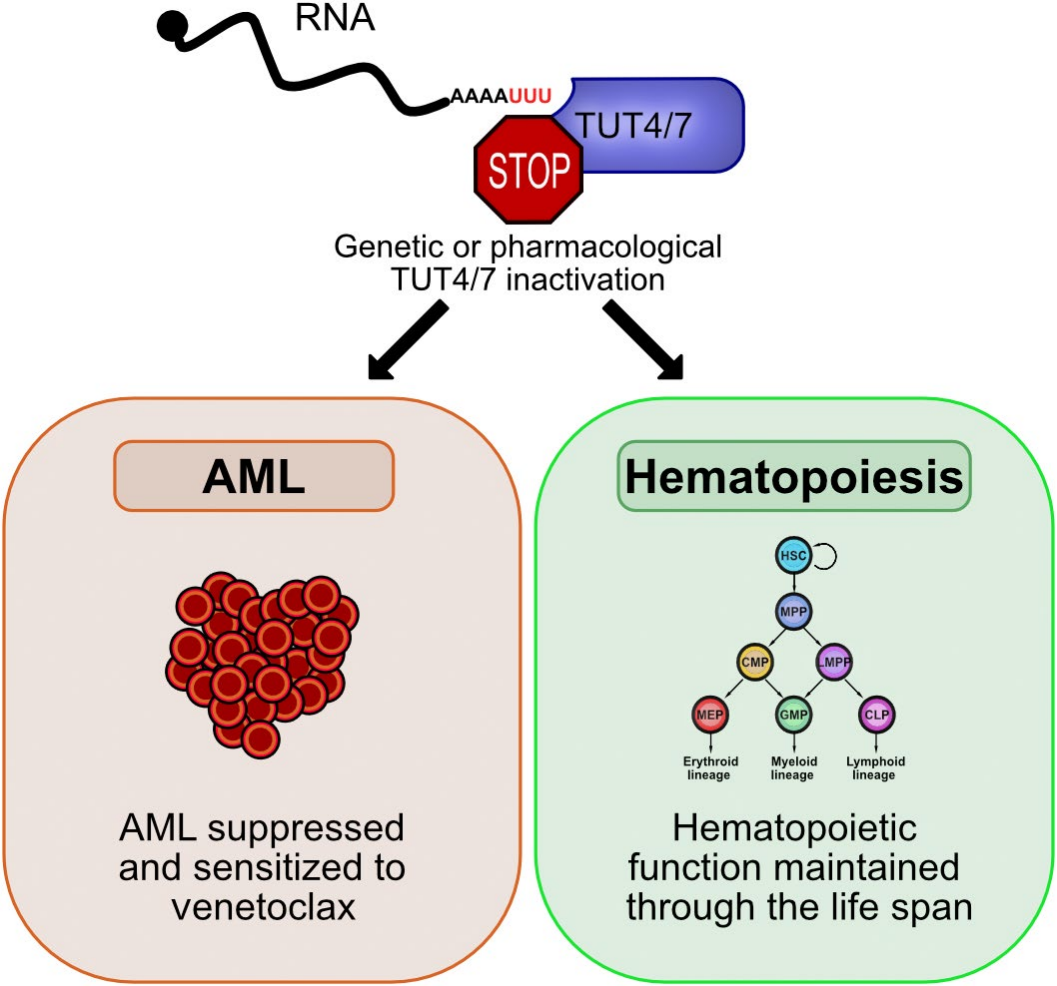
Graphical abstract. Genetic or pharmacological TUT4/7 inactivation in AML disrupts expression of mevalonate pathway genes and compromises AML cell growth and survival while synergizing with venetoclax to induce substantial cytotoxicity. Complete loss of *Tut4/7* expression does not derail steady-state hematopoiesis but causes mild inflammatory activation resulting in age-associated HSPC expansion and myeloid biased differentiation.

Our findings implicate RNA uridylation as a key posttranscriptional modification in cancer pathogenesis in vivo, positioning RNA uridylyl transferases alongside RNA methyltransferases as a new class of therapeutic targets in blood malignancies (*14*, *15*). Genetic depletion of *Tut4/7* via knockdown or knockout slows leukemic cell proliferation, induces their apoptosis, and extends survival of in vivo models. Pharmacological TUT4/7 inhibition using the preclinical compound 2266 curtails growth and survival of AML cells in vitro and sensitizes patient samples to venetoclax. Mechanistically, both genetic and pharmacological TUT4/7 inactivation converge on metabolic reprograming, most prominently through down-regulation of genes involved in the mevalonate pathway. Consistent with this model, inhibition of cholesterol synthesis and prenylation synergizes with 2266, substantially affecting AML cell proliferation (Fig. 9).

We previously demonstrated that TUT4/7 inhibitor 2266 substantially diminishes miRNA uridylation (*41*). The concordance in phenotypic and molecular impacts of TUT4/7 pharmacological inhibition and genetic ablation demonstrated by this study is consistent with on-target activity.

Despite high expression within HSPCs, genetic *Tut4/7* ablation is compatible with hematopoietic function and mice survive for at least 52 weeks without obvious adverse phenotypes. While TUT4/7 are dispensable for HSC function, they are required to suppress inflammatory activation in downstream progenitor cells, protecting the hematopoietic system from aberrant expansion with age. We find no evidence that *Tut4/7* inactivation results in bone marrow failure, fibrosis, or malignancy. Thus, we report therapeutic targeting of TUT4/7 as a selective strategy against AML, which is well tolerated by the hematopoietic system. Notably, metabolic dysregulation resulting from TUT4/7 inactivation was found exclusively in malignant hematopoiesis but was unaffected in normal hematopoietic *Tut4/7^cKO^* cells (fig. S4E), which may explain the selective impact of TUT4/7 inactivation on AML. Although a direct causal link connecting TUT4/7-mediated RNA degradation with mevalonate pathway gene expression remains to be fully established, our results suggest a multifaceted mechanism, potentially involving both direct regulation of metabolic transcript degradation and indirect regulation via miRNA uridylation.

Given the limited efficacy and toxicity of current AML therapies (*4*, *5*), our identification of TUT4/7 as druggable therapeutic targets holds considerable translational potential. Metabolic reprograming has emerged in recent years as a central axis of AML chemotherapy resistance (*46*, *47*, *112*). In particular, targeting of cholesterol synthesis is of growing interest for the treatment of myeloid malignancies (*88*, *91*, *92*), with reports indicating that cholesterol synthesis and prenylation are required for leukemic propagation (*42*, *44*, *45*, *87*). AML cells up-regulate mevalonate pathway activity as a survival response upon treatment with chemotherapy (*42*), and clinical trials are ongoing to combine statins with venetoclax (*91*, *92*).

Therefore, our finding that TUT4/7 are required to maintain the expression of key enzymes within metabolic pathways, including cholesterol biosynthesis and fatty acid metabolism, is of crucial translational relevance. TUT4/7 inhibition could help block this adaptive resistance mechanism, allowing selective and nontoxic targeting of AML. Thus, TUT4/7 inhibitors provide a previously unexplored strategy, both alone and to enhance the efficacy of venetoclax. Because TUT4/7 inactivation down-regulates mevalonate pathway genes selectively, this strategy could avoid sensitizing normal cells to venetoclax, thus resulting in lower toxicity than statin-mediated mevalonate blockade.

In conclusion, our findings support the development of a clinical-grade TUT4/7 inhibitor as a next-generation therapeutic strategy in AML, singly or in combination with venetoclax. Such a compound may not only enhance current standard-of-care regimens but also provide a nontoxic approach to target leukemic cells while sparing normal hematopoiesis.

## MATERIALS AND METHODS

### Experimental design

Given the successful therapeutic targeting of the m^6^A RNA modification pathway in AML, the objective of this study was to determine whether the analogous RNA uridylation pathway is similarly required for AML cell growth and survival. Because m^6^A pathway inhibition is associated with hematopoietic toxicity, we also investigated the physiological consequences of genetic deletion of the uridylation enzymes TUT4 and TUT7 in normal hematopoiesis.

This was an experimental study. We used genetic deletion, genetic KD, and pharmacological inhibition to assess the requirement for TUT4/7 activity in mouse AML models, human leukemic cell lines, and samples from patients with primary AML, both in vivo and in vitro. To evaluate the impact of TUT4/7 loss on normal hematopoiesis, we analyzed the composition and function of the hematopoietic system in mice with genetic deletion of Tut4/7.

RNA-seq, proteomics, SLAM-seq, sRNA-seq, and metabolomics were performed to identify molecular mechanisms by which TUT4/7 loss affects normal and malignant hematopoietic cells. Key findings were functionally validated in leukemic cells using a range of in vitro assays, including those probing the interaction between TUT4/7 inactivation and mevalonate pathway blockade. In the normal hematopoietic system, additional assays were used to confirm aberrant activation of pro-inflammatory signaling pathways.

Reagents, suppliers, catalog numbers, and RRIDs are provided in table S4. Where possible, primary researchers were blinded to experimental variables. In vitro experiments, *Meis1/Hoxa9* transplants, and HSC transplants were replicated independently. Analysis of normal hematopoiesis contains data generated from multiple independent experiments. Except for in vitro studies with THP-1 and NOMO-1 cells, all experiments used biological replicates generated from independent animals or embryos.

### Murine in vivo models

In vivo experiments were performed under UK Home Office authorization under project license PP4153210 at the University of Edinburgh, Barts Cancer Institute–Queen Mary University of London, and the Institute of Cancer Research, London following national and institutional guidelines. All mice were on the C57BL/6 genetic background. *Tut4^fl^, Tut7^fl^, Tut4^GFP^*, *Tut7^GFP^*, and *Vav-iCre* mice and protocols for their genotyping have been described previously (*30*, *31*, *52*). All transgenic mice were CD45.2^+^, and congenic recipient mice were CD45.1^+^/CD45.2^+^. Mice donating support/competitor bone marrow were CD45.1^+^. *Vav-iCre* and NBSGW mice were purchased from the Jackson Laboratory (*113*). Mice were randomized within cages, and wherever possible, mice were age and sex matched.

Sample sizes for all experiments were informed by prior studies. For leukemic transplantation on the basis of prior effect sizes and empirical experience with leukemia transplantation assays, cohort sizes of approximately 8 to 10 mice per group are sufficient to detect biologically meaningful differences in survival. To account for variability in engraftment efficiency, disease latency, and experimental attrition inherent to in vivo leukemia models, leukemic experiments were therefore performed using 10 mice per group.

In contrast, genetic or pharmacological perturbation of RNA modification pathways has previously been associated with relatively modest effects on normal hematopoiesis. To ensure sufficient sensitivity to detect subtle but biologically relevant changes in hematopoietic composition and function, larger cohorts were used for transplantation experiments assessing normal hematopoiesis. Group sizes of 12 to 14 mice were selected based on empirical experience with similar hematopoietic transplantation and phenotyping studies, allowing for robust statistical comparison while accommodating biological variability.

### Human tissue

Use of human tissue was in compliance with the ethical and legal framework of the UK’s Human Tissue Act, 2004. Primary human AML samples were sourced from the Barts Cancer Institute Biobank and provided with approval of the Research Ethics Committee. Use of AML samples was authorized following ethical review by the Barts Cancer Institute Tissue Biobank scientific subcommittee with the informed consent of donors. For all samples, the Barts Cancer Institute Biobank obtained informed consent from all participants.

### Murine sample preparation

To generate single-cell suspensions, bone marrow (BM) cells were isolated by crushing tibias and femurs using a pestle and mortar in cell suspension media [phosphate-buffered saline (PBS), 2 mM EDTA, and 2% fetal bovine serum (FBS)]. Splenic and fetal liver samples were prepared, by crushing samples through a 70-μm cell strainer in cell suspension media. Blood was collected following tail vein pinprick into EDTA-coated tubes. Complete blood counts and white blood cell counts to determine organ cellularity were determined using a Nihon Kohden Celltac blood counter. For select assays, erythrocytes were lysed using ammonium chloride solution (STEMCELL Technologies 07850). For transplants and other select assays, red blood cell–lysed single-cell suspensions were enriched for c-Kit expression using c-Kit (CD117) MACS enrichment (Miltenyi Biotec 130-091-224).

### Flow cytometry and cell sorting

Cell surface phenotypes used to identify various hematopoietic subsets are described in table S5. To analyze HSCs and progenitor cells, following incubation with Fc block, single-cell suspensions of unfractionated BM cells were stained with lineage markers containing biotin-conjugated anti-CD4, anti-CD5, anti-CD8a, anti-CD11b, anti-B220, anti–Gr-1, and anti-Ter119 antibodies together with BV711-conjugated anti–c-Kit, APC-Cy7–conjugated anti–Sca-1, APC-conjugated anti-CD48, PE-Cy7–conjugated anti-CD150, fluorescein isothiocyanate (FITC)–conjugated anti-CD34, and PE-conjugated anti-CD135 (Flt3) antibodies. Biotin-conjugated antibodies were then stained with PerCP-conjugated streptavidin. To distinguish CD45.2^+^ donor–derived cells in the peripheral blood (PB) or BM of transplanted mice, BV711-conjugated anti-CD45.1 and Pacific-Blue–conjugated anti-CD45.2 antibodies were used. For HSC and progenitor staining in transplanted mice, APC-conjugated anti–c-Kit was used; the remainder of the staining was as described above.

For analyses of differentiated cells, single-cell suspensions were stained with APC-conjugated anti-CD11b and PE-Cy7–conjugated anti–Gr-1 for myeloid cells; PerCP-conjugated anti-B220 and APC-Cy7–conjugated anti-CD19 for B cells; and PE-conjugated anti-CD4 and anti-CD8 antibodies for T cells. For analyses of differentiated cells in PB and BM of transplanted mice, myeloid cells and lymphoid cells were stained as described above with the addition of BV711-conjugated anti-CD45.1 and Pacific Blue–conjugated anti-CD45.2 antibodies.

TO-PRO-3 or 4′,6-diamidino-2-phenylindole (DAPI) was used for dead cell exclusion. Flow cytometry analyses were performed using an LSRFortessa (BD Biosciences), BD FACSymphony A3, or Agilent NovoCyte Penteon. Cell sorting was performed on a FACSAria Fusion (BD Biosciences) or BD FACS ARIA II.

To analyze intracellular protein phosphorylation, c-Kit–enriched single-cell suspensions were incubated with Fc block and stained with biotin-conjugated anti-Lineage marker antibodies (anti-CD4, anti-CD5, anti-CD8a, anti-CD11b, anti-B220, anti-Gr-1, and anti-Ter119), APC-Cy7–conjugated anti–c-Kit, and Pacific Blue–conjugated anti–Sca-1. Biotin-conjugated Lineage markers were then stained with PerCP-conjugated streptavidin. Cells were then fixed and permeabilized using Phosflow Lyse/Fix, Phosflow Perm Buffer III, and stain buffer (all from BD Biosciences) according to the manufacturer’s instructions. After processing, cells were stained with AF647-conjugated anti-pStat3.

### Leukemic transformation

To prepare *Meis1/Hoxa9* leukemic cells, c-Kit^+^ cells were prepared from fetal livers of E14.5 embryos using c-Kit (CD117) MACS enrichment (Miltenyi Biotec 130-091-224). A total of 200,000 c-Kit^+^ cells were transduced with retroviruses encoding *Meis1* and *Hoxa9.* Two thousand transformed cells were grown for 7 days in MethoCult M3231 (STEMCELL Technologies 03231) supplemented with stem cell factor (SCF; 20 ng/ml), interleukin-3 (IL-3; 10 ng/ml), IL-6 (10 ng/ml), and granulocyte-macrophage colony-stimulating factor (10 ng/ml). Using 2000 cells each time, cells were serially replated twice more into culture conditions described above to generate colony-forming leukemic cells able to initiate leukemic disease in vivo. For production of other murine leukemic cells, c-Kit^+^ single-cell bone marrow suspensions were prepared and transduced with retroviruses encoding *NRAS* and *AML-ETO* or *MLL-AF9* or *AML1-ETO*.

### Transplantation assays

CD45.1^+^/CD45.2^+^ recipient mice were lethally irradiated using a split dose of 8 Gy (two doses of 4 Gy administered at least 4 hours apart) at an average rate of 1.086 Gy min^−1^ using a RADSOURCE x-ray irradiator.

For primary transplantation of leukemic cells, 100,000 *Meis1*/*Hoxa9*-transduced c-Kit^+^ cells produced as described previously were transplanted into lethally irradiated CD45.1^+^/CD45.2^+^ recipient mice (together with 200,000 unfractionated support CD45.1^+^ wild-type BM cells). For secondary transplantations of leukemic cells, 50,000 cells collected from primary recipients were transplanted into lethally irradiated CD45.1^+^/CD45.2^+^ recipient mice (together with 200,000 unfractionated support CD45.1^+^ wild-type BM cells). Recipients were culled upon reaching their humane end point as recorded in survival curves.

For primary transplantation of normal hematopoietic stem cells, 200 CD45.2^+^ LSKCD48^−^CD150^+^ HSCs (per recipient) sorted from the BM of donor mice were mixed with 200,000 support CD45.1^+^ BM cells and transferred into lethally irradiated CD45.1^+^/CD45.2^+^ recipients. All recipient mice were euthanized and analyzed 16 to 20 weeks posttransplantation. For secondary transplantation, 3000 CD45.2^+^ LSK cells were sorted from the BM of primary recipient mice and mixed with 200,000 support CD45.1^+^ BM cells and transferred into lethally irradiated CD45.1^+^/CD45.2^+^ recipients. This process was repeated for tertiary and quaternary transplantations using 2000 and 1000 CD45.2^+^ LSK cells, respectively.

Xenotransplantation was performed by injecting 1600 THP-1 cells into the tail vein of immunodeficient NBSGW mice. Individual animals were culled upon reaching the humane end points described in our UK Home office license. Cause of death was confirmed as THP-1 leukemia by gross anatomy dissection and flow cytometric analysis.

Mice that died from non–disease-related causes (e.g., technical complications unrelated to leukemia or transplantation procedures) were excluded from analysis. No animals were excluded on the basis of disease severity or experimental outcome.

### Cell culture and in vitro *assays*

*Meis1/Hoxa9* cells were cultured at 200,000 cells/ml in Iscove’s modified Dulbecco’s medium (IMDM) supplemented with 10% FBS, SCF (40 ng/ml), IL-6 (20 ng/ml), IL-3 (20 ng/ml), penicillin, and streptomycin. For liquid culture of primary patient samples, frozen AML mononuclear cells were obtained from the Barts Cancer Institute Biobank and quickly thawed at 37°C. Samples were plated with concentrations of 0.4 × 10^6^ to 1.0 × 10^6^ ml^−1^ in StemSpan SFEM medium (STEMCELL Technologies 09600) supplemented with 2.5% Myelocult H5100 (STEMCELL Technologies 05150), and IL-3, IL-6, SCF, and FLT3L (10 ng ml^−1^; PeproTech). To perform drug assays, media was supplemented with compounds at concentrations described for each assay or dimethyl sulfoxide (DMSO) vehicle control at an identical concentration. TS-002266 was sourced from Redona Therapeutics, and other targeted inhibitors were sourced from MedChemExpress.

To perform assays of normal hematopoietic CFCs, unfractionated BM cells were cultured in MethoCultTM M3434 methylcellulose medium (STEMCELL Technologies 03434). Two technical replicates were used for each biological replicate and colonies were categorized and enumerated on day 10 of growth.

AML patient sample CFC assays were conducted by plating 1.5 × 10^5^ human primary AML cells in MethoCult H4434 (STEMCELL Technologies 04434) methylcellulose medium with either 10 μM 2266 or DMSO vehicle control. After 11 days, colonies were enumerated. Two/three technical replicates were used per biological replicate.

All primary and established cell lines were mycoplasma tested and confirmed free from contamination. Human cell lines were purchased from the American Type Culture Collection or DSMZ. *Npm1;Flt3-ITD* and *Mll-Af9;Flt3-ITD* leukemic cells (*53*, *54*) were provided by G. S. Vassiliou.

### shRNA-mediated *TUT4/7* KD

THP-1 and NOMO-1 cells were transduced with lentiviruses expressing shRNAs: [*shRNA-Tut4* (*1*): 5′-CCGGGCTTCTGACCTTAATGATGATCTCGAGATCATC ATTAAGGTCAGAAGCTTTTTTG-3′], [*shRNA-Tut4* (*2*) 5′-CCGGGCAACAGACATGTACAGATAACTCGAGTTATCTGTACATGTCTGTTGCTTTTTTG-3′], [*shRNA-Tut7* (*1*) 5′-CCGGCCAAGAGAAACGCCGATTAAACTCGAGTTTAATCGGCGTTTCTCT TGGTTTTTTG-3′], [*shRNA-Tut7* (*2*) 5′-CCGGCCAAGAGAAACGCCGATTAAACTCGAGTTTAATCGGCGTTTCTCTTGGTTTTTG-3′], and (*shRNA-*scrambled control: 5′-CGGTCCATTAATAACTATAAC-3′).

### RT-qPCR

The High-Capacity cDNA Reverse Transcription Kit was used for all reverse transcription quantitative polymerase chain reaction (RT-qPCR) reactions (Thermo Fisher Scientific—4368813), with 1 μg of RNA per reaction. Technical triplicates for each sample were analyzed. qPCR analysis was performed using MicroAmp Optical 384-Well Reaction Plates (Thermo Fisher Scientific—4343370), 2.5 μl of TaqMan Universal PCR Master Mix (Thermo Fisher Scientific—4304437), 0.25 μl of TaqMan probe/primer set for each gene (namely Hprt1 Hs02800695_m1; TUT4 Hs01118545_m1; TUT7 Hs00612265_m1), 1.25 μl of distilled water, and 1 μl of cDNA. All signals were quantified using the 2^−ΔΔCt^ method using Hprt1 as the housekeeping gene for normalization.

### Cell proliferation and cell death analyses

To determine proliferation, viable cells were counted either manually using Trypan blue exclusion or using DAPI exclusion using an Agilent NovoCyte Penteon. To analyze cells undergoing apoptosis, cells were suspended in binding buffer containing PE-conjugated Annexin V or FITC-conjugated Annexin V and either PI or DAPI before flow cytometric analysis.

### Drug synergy assays

Drug synergy assays were performed in high throughput using the CellTiter-Fluor Cell Viability Assay (Promega G6082). CellTiter assays were performed according to the manufacturer’s instructions. Briefly, cells were grown in a matrix of concentrations representing two drugs within opaque 96-well tissue culture plates. CellTiter-Fluor reagents were resuspended, mixed, and added to cells at a 1:1 ratio before mixing and incubation for 30 min. The number of viable cells was then read out using an HDSD Pherastar 1 microplate reader. Fluorescence readings were blanked by subtracting the value gained from wells without cells containing the maximum drug concentrations. The percentage inhibition for each drug combination was then calculated by determining the % growth decrease in treated versus vehicle control wells. Synergy was calculated using the Synergy Finder+ web application (*114*) using the Bliss model without baseline correction.

### Cytokine profiling

Cytokine profiling was performed using the LEGENDplex MU Cytokine Release Syndrome Panel (13-plex) kit (BioLegend 741024) according to the manufacturer’s instructions using blood plasma on a BD Fortessa Flow cytometer.

### Bulk RNA extraction, sequencing, and bioinformatic analysis

All RNA-seq library preparation was performed using rRNA depletion not poly-A enrichment-based protocols to avoid aberrant results arising from tail length/modification. For sequencing of 2266 or vehicle control–treated *Npm1/Flt3-ITD* and *Mll-AF9/Flt3-ITD* cells, total RNA was extracted from 1,000,000 cells per replicate using Direct-zol Miniprep kits (Zymo Research R2052) following the manufacturer’s protocol. The RNA integrity number (RIN) was determined by High Sensitivity RNA ScreenTape (Agilent, catalog no. 5067) and all RIN was >8. Qubit RNA High Sensitivity assay was used to determine RNA concentration. Six hundred nanograms per sample was input into rRNA depletion RiboCop (HMR) V2 RNA-seq library preparation using Lexogen CORALL Total RNA-Seq V2 with UDI set B1 (Lexogen). The PCR add-on kit (Lexogen) was used to determine PCR cycle number for library amplification. Paired-end library sequencing was performed using a Novaseq X plus by Novogene to generate 40M reads per sample. Sequencing of 2266-treated *Meis1/Hoxa9* cells was performed as above, but library preparation and sequencing was performed on 600 ng per sample submitted to Novogene for lnc-RNA-seq. For analysis of differentially expressed genes, reads were subjected to trimming with trim-galore (v.0.6.5) to remove adapter sequences and low-quality reads with the following parameters: --paired –retain_unpaired –illumina –gzip. Reads were aligned to the mouse genome (mm39) with the STAR aligner (v.2.7.9a). Reads mapped to exons were counted with featureCounts (version 2.0.dddc6) using the ENSEMBL v110 annotations. Differentially expressed genes were analyzed using DESeq2 (version 1.42.1).

For sequencing of *Tut4/7^CTL^* and *Tut4/7^cKO^ Meis1/Hoxa9* cells, total RNA was extracted from 1,000,000 preleukemic cells using RNeasy Plus Universal Mini kit (QIAGEN, catalog no. 73404) following the manufacturer’s protocol. The RIN was determined by High Sensitivity RNA ScreenTape analysis (Agilent, catalog no. 5067) and confirmed to be >8. RNA-seq libraries were prepared from 1 μg of total RNA using the NEBNext Ultra II Directional RNA Library Prep kit for Illumina (NEB, catalog no. E7760) with the NEBNext rRNA Depletion Kit (Human/Mouse/Rat) (NEB, catalog no. E6310) following the manufacturer’s protocol. Eight cycles of PCR were performed to amplify libraries, and the pooled library was sequenced on Illumina NextSeq 550 with 85-bp single end at the EMBL GeneCore facility. For analysis of differentially expressed genes, reads were mapped to GRCm38 genome_tran (release 84) with HISAT2 (2.1.0) after trimming adapter sequences using cutadapt (1.17). Mapped reads per gene were counted with htseq-count (HTSeq 0.11.1) providing GTF file, and differentially expressed genes were analyzed using DESeq2.

Gene enrichment analyses were performed with the clusterProfiler package (v4.10.1) in R (v4.3.2) with 1000 permutations and default parameters. PANTHER Classification System (*115*) was used to conduct GO statistical overrepresentation test against the mouse coding genome using Fisher’s exact test with Benjamini-Hochberg FDR correction.

### Single-cell RNA-seq

10x Genomics processing and analysis was performed as described previously (*116*). LK and LSK cells were isolated from two wild-type and two knockout donors. A total of 20,000 cells per sample were sorted by flow cytometry, centrifuged, and resuspended in PBS + 0.04% bovine serum albumin and immediately processed with the 10x Genomics Single Cell 3′ v3 protocol following the manufacturer’s instructions. Libraries were sequenced using the Illumina NovaSeq instrument, obtaining at least 30,000 reads per cell in each run. 10x Genomics reads were preprocessed using cellranger (version 3.1.0, reference genome and annotation version 3.0.0) with default settings. Downstream analysis was performed mainly using the scanpy framework. Low-quality barcodes with less than 2100 genes per cell and 3000 RNAs were excluded from the analysis, doublet scores were estimated using the scrublet tool (using 30 principal components), and potential doublets were removed (top 4% in each sample). One sample was excluded from the analysis due to low quality. Data were normalized to 10,000 total counts and ln(*n* + 1) transformed. A landscape containing all samples was generated by selection of highly variable genes, principal components analysis calculation, nearest-neighbor graph estimation, Leiden clustering, and UMAP projection. Genes correlated with cell cycle were excluded. The residual cell cycle was regressed out using a linear model. For differential expression, cells were pseudobulked per Leiden cluster and per sample and analyzed using edgeR. Ingenuity pathway analysis was performed on pseudobulked data using the Core Analysis Function offered by QIAGEN’s Ingenuity Pathway Analysis software. The interrogated RNA-seq and MS datasets were filtered for adjusted *P* values of differential expression (FDR < 0.05), and the threshold for significant activation or inhibition was defined by an absolute *z*-score > 2.

### Proteomics

Cell pellets were lysed in a buffer containing 1% sodium deoxycholate (SDC), 100 mM triethylammonium bicarbonate, 10% isopropanol, and 50 mM NaCl, freshly supplemented with 5 mM TCEP (Thermo Fisher Scientific, Bond-breaker), 10 mM iodoacetamide, universal nuclease 1:2000 vol/vol (Pierce, no. 88700) and Halt protease and phosphatase inhibitor cocktail (Thermo Fisher Scientific, no. 78442, 100×) with 5 min of bath sonication. Protein concentration was measured with the Quick Start Bradford protein assay (Bio-Rad). Aliquots of 30 μg of total protein were digested overnight with trypsin (Pierce, 1:20) at room temperature. Peptides were labeled with the TMTpro reagents (Thermo Fisher Scientific) by adding 5 μl of the reagent (25 μg/μl) into 12.5 μl of sample volume. The TMTpro mixture was acidified with formic acid at 2% and the precipitated SDC was removed by centrifugation. The peptide pool was fractionated with high-pH reversed-phase chromatography using the Xbridge C18 column (2.1 mm by 150 mm, 3.5 μm, Waters) on an UltiMate 3000 HPLC system over a 1% gradient in 35 min. Mobile phase A was 0.1% (v/v) ammonium hydroxide and mobile phase B was 0.1% ammonium hydroxide (v/v) in acetonitrile.

Liquid chromatography-mass spectrometry (LC-MS) analysis was performed on a Vanquish Neo UHPLC system coupled to the Orbitrap Ascend mass spectrometer (Thermo Fisher Scientific) using a 25-cm capillary column (Waters, nanoE MZ PST BEH130 C18, 1.7 μm, 75 μm by 250 mm) over a 100-min gradient 5 to 35% of mobile phase B composed of 80% acetonitrile and 0.1% formic acid. Peptides were preconcentrated onto a PEPMAP 100 C18 5-μm 0.3 mm by 5 mm 1500 Bar trapping column and the analytical column was connected to a stainless steel emitter on the Nanospray Flex ion source. MS spectra were collected at an Orbitrap mass resolution of 120,000 and precursors were selected for higher energy collisional dissociation (HCD fragmentation) in the top speed mode (3 s) with collision energy 32% and iontrap detection in turbo scan rate. MS3 scans were triggered by Real Time Search (RTS) against a fasta file containing UniProt *Mus musculus* reviewed canonical and isoform sequences with Synchronous Precursor Selection isolation (10 notches) and HCD fragmentation with collision energy 55% at 45,000 Orbitrap resolution. Selected precursors were dynamically excluded from further activation for 45 s with 10 parts per million (ppm) mass tolerance and RTS close-out was enabled with a maximum of four peptides per protein. Static modifications for RTS were TMTpro16plex at K/n-term (+304.2071) and Carbamidomethyl at C (+57.0215), and variable modifications were Deamidated NQ (+0.984) and Oxidation of M (+15.9949) with maximum one missed cleavage and two variable modifications per peptide. An additional injection of each fraction was conducted by including Farnesyl (+204.188 Da) and GeranylGeranyl (+272.250 Da) at C, as well as Carbamidomethyl at C (+57.0215) as variable modifications in the RTS analysis.

The Sequest HT and Comet nodes in Proteome Discoverer 3.0 (Thermo Fisher Scientific) were used to search the raw mass spectra against a fasta file containing reviewed UniProt *M. musculus* entries. The precursor mass tolerance was set at 20 ppm, and the fragment ion mass tolerance was set at 0.5 Da (or 1 Da for Comet) with up to two trypsin missed cleavages allowed. TMTpro at N terminus/K were defined as static modifications. Dynamic modifications were Oxidation of M (+15.995 Da), Deamidation of N/Q (+0.984 Da), Farnesyl (+204.188 Da) at C, GeranylGeranyl (+272.250 Da) at C, and Carbamidomethyl (+57.0215) at C. Peptide confidence was estimated with the Percolator node and peptide FDR was set at 0.01 based on target-decoy search. Only unique peptides were used for quantification, considering protein groups for peptide uniqueness. Peptides with average reporter signal-to-noise ratio greater than 3 were used for protein quantification. The mass spectrometry proteomics data have been deposited to the ProteomeXchange Consortium via the PRIDE (*117*) partner repository with the dataset identifier PXD077084

### SLAM-seq

SLAM-seq libraries were prepared using the Lexogen catabolic kit (catalog no. 062.24) and the Lexogen QuantSeq 3′ mRNA-Seq Library Prep Kit FWD for Illumina (catalog no. 015.24) in both cases following the manufacturer’s instructions. For labeling cells, 4-thiouridine (S4U) was used at 2.9 μM, as determined by the cell viability titration assay previously described (*9*). Medium with 4SU was used for preleukemic stem cells labeling for 12 hours and was later replaced with 4SU-free medium (time 0). Cells were collected into 1 ml of QIAzol reagent immediately after changing medium and at 1, 3, and 9 hours and frozen on dry ice. Total RNA was extracted and alkylated following the manufacturer’s instructions. Total RNA (500 ng) was used for library preparation and 16 PCR cycles were performed. Pooled 24 libraries were sequenced using an Illumina NextSeq 500 in 85-bp single-end mode. Biological triplicates for both *Tut4/7^CTL^* and *Tut4/7^cKO^* preleukemic cells were used to generate the different library sets. SLAM-seq libraries were analyzed as described previously (*56*). Briefly, T-to-C conversion rates were obtained using the SlamDunk (v 0.4.3) pipeline. Conversion rates across different time points were normalized to time 0 for each gene. Genes that have mean normalized value >0.8 and SD <0.3 at time 0 were used for further analyses to calculate half-lives by fitting to an exponential decay model.

### sRNA-seq

Small RNAs were size selected from 1 μg of total RNA and libraries were prepared using the NEBNext small RNA library prep kit (E7330). Sixteen cycles of PCR were performed to amplify libraries and PCR product was size selected on acrylamide gel. Purified libraries were pooled and sequenced on Miniseq with single-ended mode 51 nt. Adapter sequences were removed using cutadapt (*118*). Trimmed reads were mapped to modified mature miRNA reference sequences retrieved from miRbase (*119*) using bowtie without allowing any mismatches. Three Ts were added to the 3′ end of reference sequences to allow any mono/poly-uridylated miRNAs to be mapped. Raw read count was normalized and compared using Deseq2 (1.42.1). Any query miRNAs that have additional T(s) at the 3′ end were counted as uridylated by an in-house pipeline and these counts were used for further analyses.

### Targeted metabolomics

Cholesterol and cholesteryl esters were measured according to the protocol published by Chandramouli and Kamat (*120*). Cells from four independent biological replicate *Meis1/Hoxa9* transformed leukemic cell lines were seeded at 400,000/ml in 5 μM 2266, 1 μM simvastatin, or DMSO vehicle control. After 48 hours, 10 million cells were harvested. Pellets were resuspended in 1 ml of PBS and transferred into glass vials, and 25 μl was aliquoted for protein normalization using BCA. Then, lipid extraction was performed by the addition of 3 ml of 2:1 (v:v) chloroform/methanol containing 1 nmol of C17:0 cholesteryl ester (Avanti Polar Lipids 700186 M) and 2 nmol of cholesterol-d7 (Avanti Polar Lipids 700041P). Samples were vortexed and centrifuged at 2000 rpm for 15 min before the bottom organic layer was collected, transferred into a clean glass vial, and dried under a nitrogen stream. Lipid extracts were then resuspended in 200 μl of 2:1 (v:v) chloroform/methanol and transferred into glass LC-MS vials for analysis. Metabolite quantification was achieved using a UHPLC-Q/TOF system. Briefly, chromatographic separation was carried out on an Agilent 1290 Infinity II Binary Pump using a Gemini 5U C18 column (5 μm, 50 mm by 4.6 mm) (Phenomenex 00B-4435-E0) maintained at 40°C. Samples were kept at 10°C in the autosampler, and the injection volume was 10 μl. Metabolites were eluted at a flow rate of 0.5 ml/min with solvent A (H_2_O:MeOH, v:v) + 0.1% formic acid +10 mM ammonium formate and solvent B (80:20 IPA:MeOH, v:v) + 0.1% formic acid +10 mM ammonium formate. The following gradient was used for elution: 0 to 4 min, 45% B; 4 to 6 min, 40 to 60% B; 6 to 16 min, 60 to 100% B; 16 to 22 min, 100% B; 22 to 24 min, 100 to 40% B; and 24 to 30 min, 40% B. Mass spectrometry analysis was performed on an Agilent 6546 LC/Q-TOF mass spectrometer operated in positive ion mode. Instrument parameters were as follows: gas temperature, 250°C; gas flow, 10 liters/min; nebulizer pressure, 45 psi (310.3 kPA); sheath gas temperature, 250°C; sheath gas flow: 10 liters/min; capillary voltage, 3000 V; fragmentor voltage, 80 V; and nozzle voltage, 1000 V. Data acquisition was performed using Agilent MassHunter Workstation software (Agilent M5960AA). Data analysis was carried out by creating a Personal Compound Database Library specific to the Cholesterol and Cholesteryl ester species detected using MassHunter PCDL Manager, followed by targeted feature extraction using Agilent Profinder B.8.0.00 (Agilent G3835AA). Data are deposited in metabolomics workbench (*121*)

### Statistical analysis and use of open-source data

Unless otherwise stated, statistical analyses were performed using GraphPad Prism v.10 software (GraphPad Software). Study design was based on experience from previous experiments, and transplantation experiments were randomized and blinded to the primary researcher. In leukemic transplants, Kaplan-Meier survival curve statistics were determined using the Log-rank (Mantel Cox) test. For normal transplants, Mann-Whitney *U* test was used to determine significant differences in hematopoietic engraftment. For in vitro assays, paired *t* tests were used when comparing biological replicates with treatment or vehicle control. For other leukemic in vitro assays, unpaired Student’s *t* test was used unless otherwise stated. For analysis of in vivo normal hematopoiesis, Mann-Whitney *U* test was used unless otherwise stated. Numbers (*n*) are provided in the figure legend for each experiment. Unless otherwise stated, data represent mean ± SEM.

Representation factor and associated probability/significance of overlap between gene lists was calculated using the hypergeometric probability test. Inputs were the total number of significantly dysregulated (FDR <0.05) transcripts in each list, overlapping significantly dysregulated genes in paired datasets and the number of protein coding genes in the mouse genome. GO analysis was performed using PantherDB version 19 using the statistical overrepresentation test—GO biological process complete. *TUT4* and *TUT7* expression in normal and malignant hematopoietic subsets was sourced from GSE63270 (*122*). Stratification of survival of patients with AML by expression of *TUT4* and *TUT7* was performed using The Cancer Genome Atlas, Leukemia, Acute Myeloid Leukemia (TGCA LAML) dataset (*123*) via Xeno Browser (*124*).
