# Supplementary material for: RNA terminal uridylyl transferases are druggable vulnerabilities in AML but are dispensable for normal hematopoiesis
Source: Sci Adv. 2026 Aug 12;12(33):eaec3399. doi: 10.1126/sciadv.aec3399 (PMC13464643; doi:10.1126/sciadv.aec3399)
Supplement: Supplementary file 1 [file sciadv.aec3399_sm.pdf]

Science Advances
AAAS

Supplementary Materials for

RNA terminal uridylyl transferases are druggable vulnerabilities in AML but are dispensable for normal hematopoiesis

Christopher Mapperley *et al.*

Corresponding author: Kamil R. Kranc, kamil.kranc@icr.ac.uk

Sci. Adv. **12**, eaec3399 (2026)
DOI: 10.1126/sciadv.aec3399

The PDF file includes:

Figs. S1 to S4
Tables S1 and S5
Legends for tables S2 to S4

Other Supplementary Material for this manuscript includes the following:

Tables S2 to S4

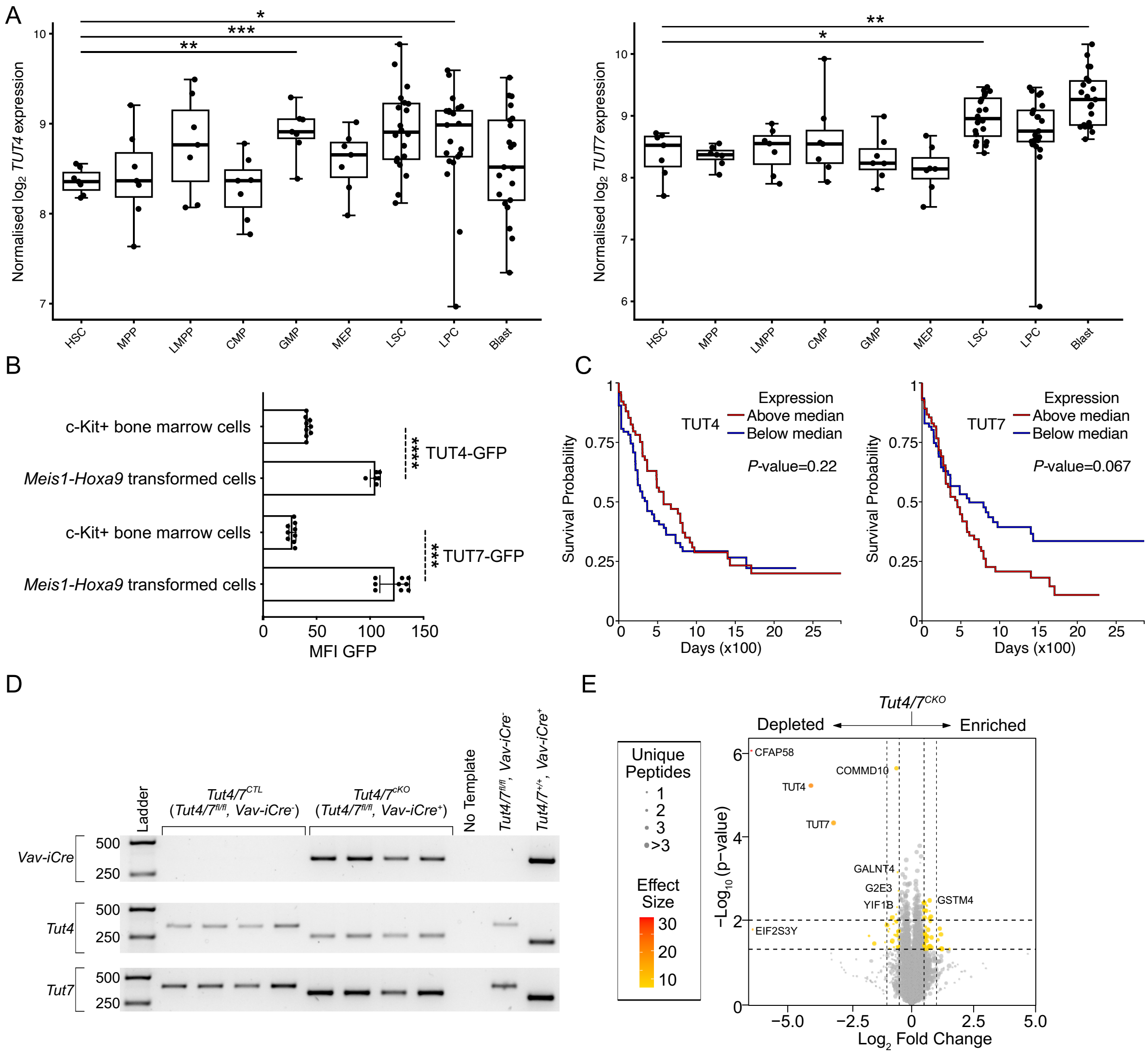

Supplemental Figure 1: *TUT4/7* expression in patient samples and knockout model validation. (A) Log_2 transformed expression of *TUT4* and *TUT7* in normal and malignant hematopoietic cells including Hematopoietic stem cells (HSC), Multipotent Progenitors (MPP), Lymphoid Primed Multipotent Progenitors (LMPP), Megakaryocyte-Erythroid Progenitor (MEP), Granulocyte-Monocyte Progenitors (GMP), Leukemic Stem Cells (LSCs), Leukemic Progenitor Cells (LPC) and Leukemic Blast cells (Blast). (Brown-Forsythe and Welch ANOVA tests, * = *P* value <0.05, ** = *P* value <0.01, *** = *P* value < 0.001), data sourced from GEO dataset GSE63270. **(B)** Expression of TUT4-GFP and TUT7-GFP fusion protein reporters in c-Kit$^+$ cells MACS enriched from *Tut4^{GFP}* and *Tut7^{GFP}* mice and in matched cells following transformation with *Meis1/Hoxa9* oncogenes (Students T Test, ***= *P* value < 0.001, ****= *P* value <0.0001). **(C)** Kaplan-Meier survival curves of overall survival from the TCGA Acute Myeloid Leukemia cohort (LAML) stratified by *TUT4* and *TUT7* expression. Data sourced and plots generated using UCSC Xena Browser. **(D)** Agarose gel electrophoresis of PCR products from *Tut4/7^{CTL}* (T*ut4$^{fl/fl}$;Tut7$^{fl/fl}$;Vav-iCre$^-$*) and *Tut4/7^{cKO}* (T*ut4$^{fl/fl}$;Tut7$^{fl/fl}$;Vav-iCre$^+$*) *Meis1/Hoxa9* transformed leukemia initiating cell biological replicates (see Figure 1H) alongside no-template (H_2O) control, and murine ear clip DNA from control T*ut4$^{fl/fl}$;Tut7$^{fl/fl}$;Vav-iCre$^-$* and T*ut4$^{+/+}$;Tut7$^{+/+}$;Vav-iCre$^+$* mice. Top gel: Vav-iCre detection PCR (*Vav-iCre$^+$*=400bp, *Vav-iCre$^-$*= no PCR

product). Middle gel: *Tut4* deletion specific PCR (*$Tut4^{fl}$= 312bp, $Tut4^{+}$*= 210bp, *$Tut4^{-}$*= 248bp. Bottom gel *Tut7* deletion specific PCR (*$Tut7^{fl}$*= 388bp, *$Tut7^{+}$*= 290bp, *$Tut7^{-}$*= 324bp). **(E)** Volcano plot showing log_2 fold change and -log_{10}(*P*-value) of protein abundance in *$Tut4/7^{cKO}$* verses *$Tut4/7^{CTL}$ Meis1/HoxA9* cells (n=4). Highlighted proteins are significantly (*P*-value<0.05) dysregulated.

H

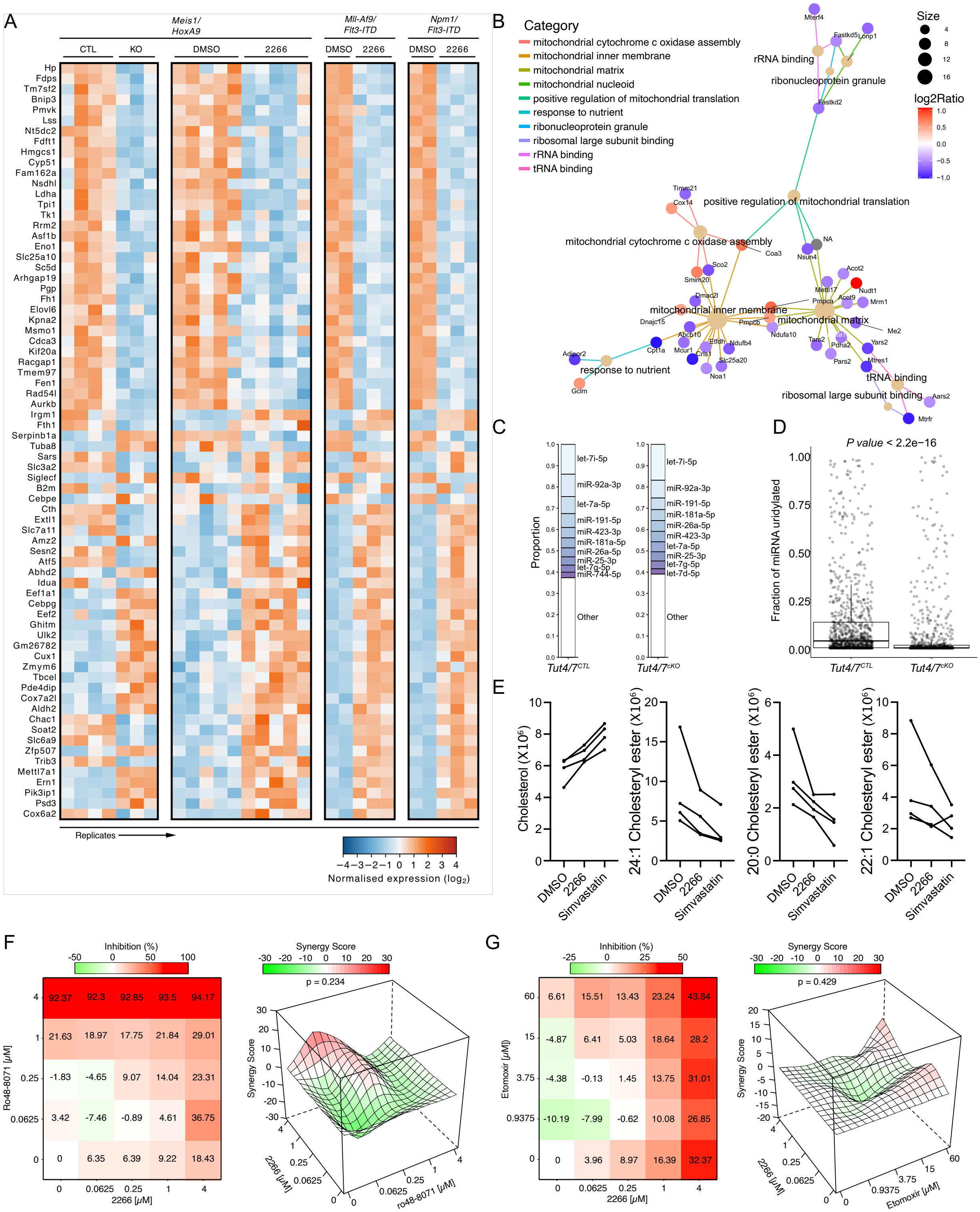

***Bcl2* expression**		FC (log2)	padj
2266	*Meis1/Hoxa9*	0.214	0.803
	MLL-AF9, FLT3-ITD	0.165	0.935
	NPM1, FLT3-ITD	0.423	0.022
Tut4/7 cKO	*Meis1/Hoxa9*	1.191	0.000

Supplemental Figure 2: TUT4/7 inactivation dysregulates metabolic pathways and drives Bcl2 overexpression. (A) Heatmap of normalized gene expression from RNA-seq for all 72 genes significantly dysregulated across all four datasets (adjusted *P*-value<0.05 in each independently). Each column represents an individual biological replicate. **(B)** Concept Network (CNET) plot showing relationships between significantly dysregulated proteins (*P*-value<0.05) and the significantly enriched gene ontology terms to which they are assigned for proteomics data described in Figure 3D **(C)** Proportion of the miRNA repertoire accounted for by the most abundant individual miRNAs vs all other miRNAs in $Tut4/7^{CTL}$ and $Tut4/7^{cKO}$ *Meis1/Hoxa9* cells, as determined by sRNA-seq. **(D)** Fraction of each miRNA that is uridylated in $Tut4/7^{CTL}$ and $Tut4/7^{cKO}$ *Meis1/Hoxa9* cells (n=4, Wilcoxon signed-rank test). **(E)** Abundance of cholesterol, 24:1 cholesteryl ester, 20:0 cholesteryl ester and 22:1 cholesteryl ester in *Meis1/Hoxa9* cells treated with DMSO vehicle control, 2266 or simvastatin. Lines connecting points indicate paired measurements from distinct biological replicates subject to different treatments. **(F)** Percentage growth inhibition matrix and synergy plot for *Meis1/Hoxa9* cells (n=3) grown in 2266 and Ro48-8071 Fumarate for 48 hours. Growth was measured using Celltitre-Fluor and results normalized to cells treated with 0 μM 2266 and 0 μM Ro48-8071 Fumarate (vehicle control). **(G)** Percentage growth inhibition matrix and synergy score plot for *Meis1/Hoxa9* cells (n=3) grown in 2266 and etomoxir for 48 hours. Growth was measured using Celltitre-Fluor and results normalized to cells treated with 0 μM 2266 and 0 μM Etomoxir (vehicle control). **(H)** Log_2 fold change, *P*-value and adjusted *P*-value for *Bcl2* expression from RNA-seq described in Figure 3A.

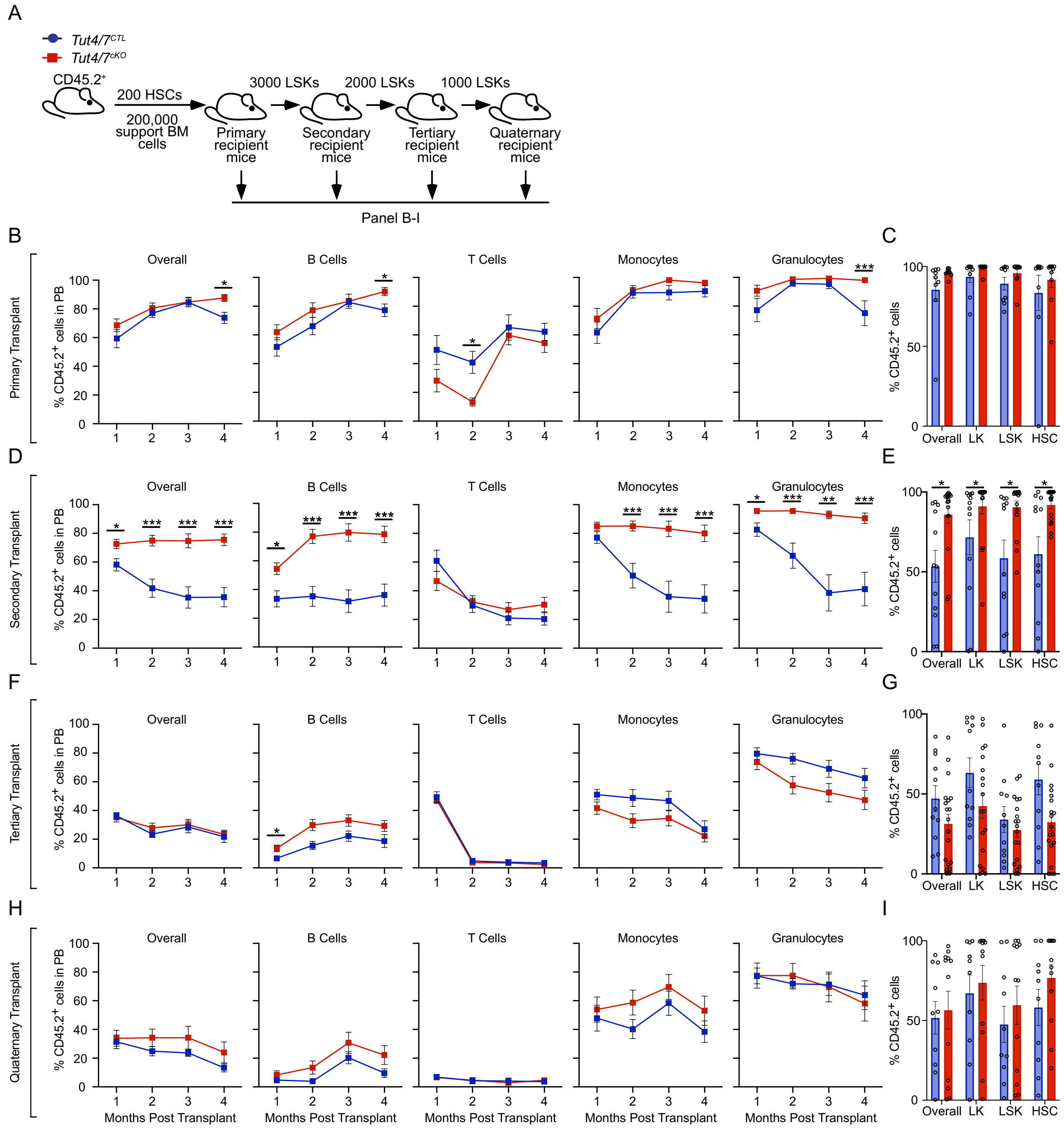

Supplemental Figure 3: Serial transplantation of *Tut4/7*^{cKO} HSPCs. (A) Experimental design, *Tut4/7*^{CTL} and *Tut4/7*^{cKO} HSCs were purified from the bone marrow and transplanted into irradiated recipient CD45.1^{+}, CD45.2^{+} mice alongside 200,000 unfractionated CD45.1^{+} competitor bone marrow support cells. Subsequently, secondary (3000 LSK), tertiary (2000 LSK) and quaternary (1000 LSK) transplantations were performed by extracting hematopoietic stem and progenitor cells (LSKs) from previous recipient mice before retransplanting to new irradiated recipients alongside bone marrow support. **(B)** Engraftment (% CD45.2/(CD45.1+CD45.2)) overall and within the B cells, T cells, Monocytes and Granulocytes in the peripheral blood of primary transplant recipient mice. **(C)** Engraftment overall and within the LK, LSK and HSC compartments within the bone marrow of primary transplant recipient mice. **(D)** Engraftment overall and within the B cells, T cells, Monocytes and Granulocytes in the peripheral blood of secondary transplant recipient mice. **(E)** Engraftment overall and within the LK, LSK and HSC compartments within the bone marrow of secondary transplant recipient mice. **(F)** Engraftment overall and within the B cells, T cells, Monocytes and Granulocytes in the peripheral blood of tertiary transplant recipient mice. **(G)** Engraftment overall and within the LK, LSK and HSC compartments within the bone marrow of tertiary transplant recipient mice. **(H)** Engraftment overall and within the B cells, T cells, Monocytes and Granulocytes in the peripheral

blood of quaternary transplant recipient mice. **(I)** Engraftment overall and within the LK, LSK and HSC compartments within the bone marrow of quaternary transplant recipient mice. Data represent mean ± SEM; *, $P < 0.05$; **, $P < 0.01$; ***, $P < 0.001$; ****, $P < 0.0001$ (Mann Whitney-U) (primary transplant n=10; secondary transplant n= CTL=12, n cKO=17; tertiary transplant n= CTL=11, n cKO=20; quaternary transplant n= CTL=10, n cKO=13).

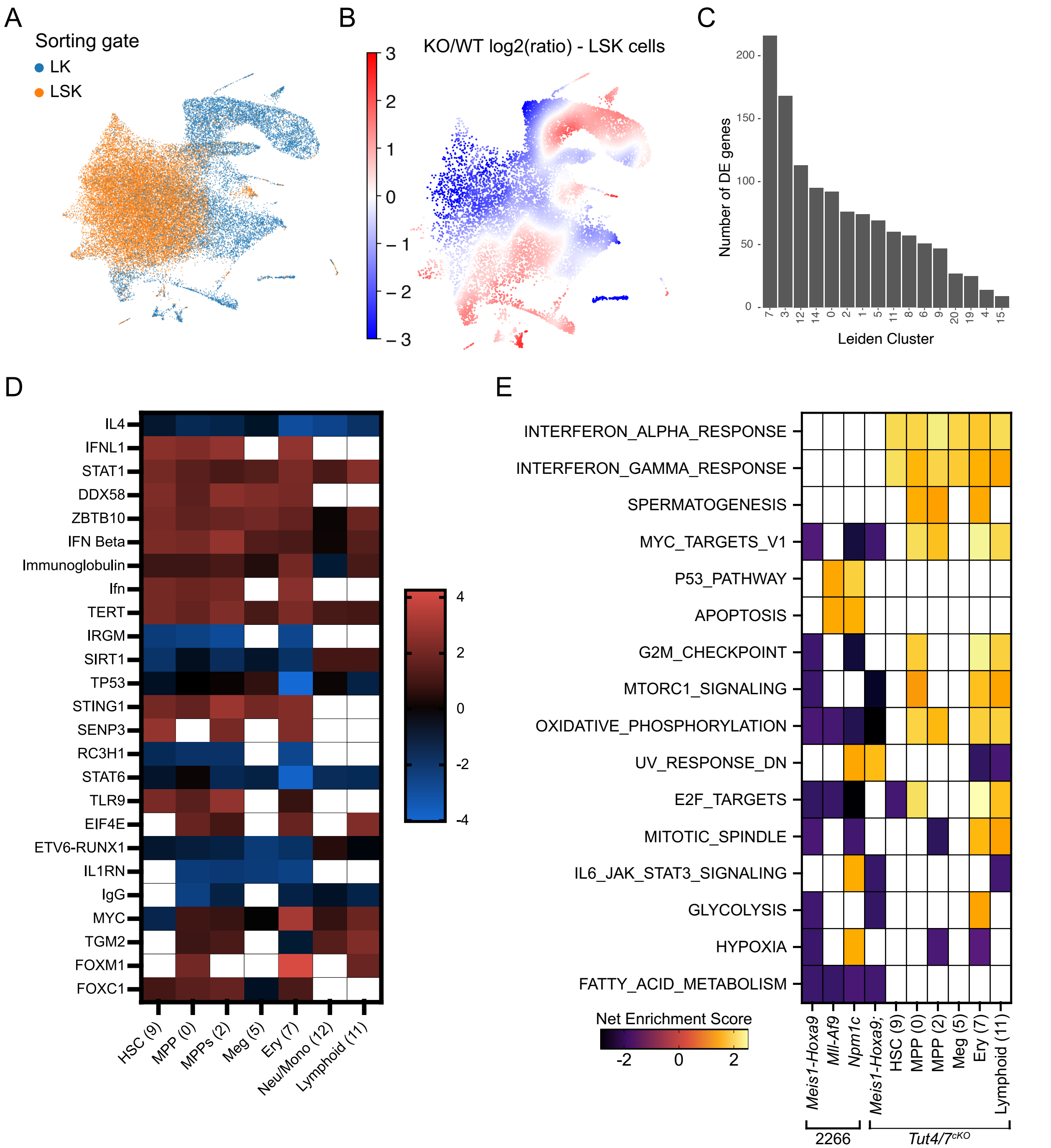

Supplemental Figure 4: TUT4/7 deficiency disrupts HSPC composition and gene expression (A) UMAP single cell RNA-seq landscape generated from sorted hematopoietic stem and progenitor cells (LSKs and LKs) indicating the location of sorted LKs (blue) and LSKs (orange). **(B)** Relative enrichment (log$_2$ ratio) of *Tut4/7*^{cKO} LK cells compared to *Tut4/7*^{CTL} LK cells representing enrichment or depletion of differential cell identities from scRNA-seq. **(C)** Number of significantly dysregulated (FDR <0.05) genes per Leiden cluster (Figure 8D). **(D)** Continuation of heatmap of z scores (Figure 8F) derived from Ingenuity Pathway Analysis (IPA) of upstream regulator analysis for Leiden clusters representing HSCs (9), MPPs (0), MPPs (2) and HSPCs biased towards megakaryocytic (5), Erythroid (7), Neutrophil/Monocyte (12) and lymphoid (11) differentiation. Pathways where expression of target genes was not detected are left blank. **(E)** Heatmap of net enrichment score for GSEA hallmarks which were significantly altered (Adjusted *P* value <0.05) in at least two RNA-seq datasets compared to vehicle or *Tut4/7*^{CTL} controls. Datasets include: *Meis1/Hoxa9*, *Mll-Af9/Flt3-ITD* and *Npm1c/Flt3-ITD cells*, treated with 2266; *Tut4/7*^{cKO} *Meis1Hoxa9 cells* and various normal

*Tut4/7^cKO^* hematopoietic subsets. White boxes represent no significant dysregulation of that pathway in that dataset.

**Representation factor of overlap**	**2266 treated *Npm1c/Flt3-ITD***	**2266 treated *Mll-Af9/Flt3-ITD***	**2266 treated *Meis1/Hoxa9***
Tut4/7^{cKO} Meis1/Hoxa9	1.7 ($P = 5*10^{-17}$)	1.7 ($P = 2.37*10^{-7}$)	1.8 ($P = 2.6*10^{-13}$)
**2266 treated *Meis1/Hoxa9***	6.5 ($P = 1.41*10^{-21}$)	12.4 ($P = 4.65*10^{-21}$)	
**2266 treated *Mll-Af9/Flt3-ITD***	24.7 ($P = 2.34*10^{-97}$)		

Supplemental Table 1: Transcriptional dysregulation resulting from genetic and pharmacological TUT4/7 inhibition significantly overlaps. Representation factor and associated significance of overlap between gene dysregulation from TUT4/7 inactivation (Figure 3A), calculated using the hypergeometric probability test. Inputs were the total number of significantly dysregulated (*P*-value <0.05) transcripts in each list, overlapping significantly dysregulated genes in paired datasets and the number of protein coding genes in the mouse genome.

Supplemental Table 2: Genetic and pharmacological TUT4/7 inactivation disrupts expression of conserved genes across AML models. Displayed are the Log2 fold change, *P*-value and FDR adjusted *P*-value for genes significantly dysregulated (*P*-value<0.05) with the direction of gene expression change consistent across all four datasets. Data also displayed in Supplemental Figure 2A.

Supplemental Table 3: Interferon alpha and gamma response genes are significantly enriched throughout the hematopoietic hierarchy. Gene set enrichment analysis (GSEA) results for HALLMARK_INTERFERON_ALPHA_RESPONSE and HALLMARK_INTERFERON_GAMMA_RESPONSE in all Leiden clusters identified from scRNA-seq of the LK and LSK compartments from *Tut4/7^{CTL}* and *Tut4/7^{cKO}* mice.

Supplemental Table 4: Reagents, materials and equipment: Contains details, catalogue numbers and RRID's for resources used by this investigation.

**Cell Subset**	**Surface Phenotype**
Lineage$^-$	CD4$^-$CD5$^-$CD8a$^-$CD11b$^-$B220$^-$Ter119$^-$Gr-1$^-$
LK	Lineage$^-$c-Kit$^+$Sca-1$^-$
LSK	Lineage$^-$c-Kit$^+$Sca-1$^+$
HSC	LSK$^+$CD48$^-$CD150$^+$
LT-HSC	LSK$^+$CD135$^-$CD34$^+$CD48$^-$CD150$^+$
MPP1	LSK$^+$CD135$^-$CD34$^+$CD48$^-$CD150$^+$
MPP2	LSK$^+$CD135$^-$CD34$^+$CD48$^+$CD150$^+$
MPP3	LSK$^+$CD135$^-$CD34$^+$CD48$^+$CD150$^-$
MPP4	LSK$^+$CD135$^+$CD34$^+$CD48$^+$CD150$^-$
CD4$^+$ T Cells	CD4$^+$
CD8$^+$ T Cells	CD8$^+$
B Cells	CD19$^+$B220$^+$
Monocytes	CD11b$^+$GR1$^-$
Granulocytes	CD11b$^+$GR1$^+$

Supplemental Table 5: Cell surface phenotypes of hematopoietic stem, progenitor and differentiated cells. Displayed are the cell surface phenotypes used to identify various hematopoietic subsets. Cell surface phenotypes of Hematopoietic stem and progenitor cells as described in Wilson et al[101].
